# Network inference reveals novel connections in pathways regulating growth and defense in the yeast salt response

**DOI:** 10.1371/journal.pcbi.1006088

**Published:** 2018-05-08

**Authors:** Matthew E. MacGilvray, Evgenia Shishkova, Deborah Chasman, Michael Place, Anthony Gitter, Joshua J. Coon, Audrey P. Gasch

**Affiliations:** 1 Laboratory of Genetics, University of Wisconsin—Madison, Madison, WI, United States of America; 2 Department of Biomolecular Chemistry, University of Wisconsin—Madison, Madison, WI, United States of America; 3 Wisconsin Institute for Discovery, University of Wisconsin–Madison, Madison, WI, United States of America; 4 Great Lakes Bioenergy Research Center, University of Wisconsin-Madison, Madison, WI, United States of America; 5 Department of Biostatistics and Medical Informatics, University of Wisconsin -Madison, Madison, WI, United States of America; 6 Morgridge Institute for Research, Madison, WI, United States of America; 7 Department of Chemistry, University of Wisconsin -Madison, Madison, WI, United States of America; 8 Genome Center of Wisconsin, Madison, WI, United States of America; University of Minnesota, UNITED STATES

## Abstract

Cells respond to stressful conditions by coordinating a complex, multi-faceted response that spans many levels of physiology. Much of the response is coordinated by changes in protein phosphorylation. Although the regulators of transcriptome changes during stress are well characterized in *Saccharomyces cerevisiae*, the upstream regulatory network controlling protein phosphorylation is less well dissected. Here, we developed a computational approach to infer the signaling network that regulates phosphorylation changes in response to salt stress. We developed an approach to link predicted regulators to groups of likely co-regulated phospho-peptides responding to stress, thereby creating new edges in a background protein interaction network. We then use integer linear programming (ILP) to integrate wild type and mutant phospho-proteomic data and predict the network controlling stress-activated phospho-proteomic changes. The network we inferred predicted new regulatory connections between stress-activated and growth-regulating pathways and suggested mechanisms coordinating metabolism, cell-cycle progression, and growth during stress. We confirmed several network predictions with co-immunoprecipitations coupled with mass-spectrometry protein identification and mutant phospho-proteomic analysis. Results show that the cAMP-phosphodiesterase Pde2 physically interacts with many stress-regulated transcription factors targeted by PKA, and that reduced phosphorylation of those factors during stress requires the Rck2 kinase that we show physically interacts with Pde2. Together, our work shows how a high-quality computational network model can facilitate discovery of new pathway interactions during osmotic stress.

## Author summary

Cells sense and respond to stressful environments by utilizing complex signaling networks that integrate diverse signals to coordinate a multi-faceted physiological response. Much of this response is controlled by post-translational protein phosphorylation. Although many regulators that mediate changes in protein phosphorylation are known, how these regulators inter-connect in a single regulatory network that can transmit cellular signals is not known. It is also unclear how regulators that promote growth and regulators that activate the stress response interconnect to reorganize resource allocation during stress. Here, we developed an integrated experimental and computational workflow to infer the signaling network that regulates phosphorylation changes during osmotic stress in the budding yeast *Saccharomyces cerevisiae*. The workflow integrates data measuring protein phosphorylation changes in response to osmotic stress with known physical interactions between yeast proteins from large-scale datasets, along with other information about how regulators recognize their targets. The resulting network suggested new signaling connections between regulators and pathways, including those involved in regulating growth and defense, and predicted new regulators involved in stress defense. Our work highlights the power of using network inference to deliver new insight on how cells coordinate a diverse adaptive strategy to stress.

## Introduction

Cells sense and respond to stressful situations by utilizing complex signaling networks that integrate diverse signals and coordinate what is ultimately a multi-faceted response. In optimal conditions, microbial cells maximize growth at the expense of stress defense by up-regulating growth related processes and suppressing defense strategies. In *Saccharomyces cerevisiae* this is mediated in part by the nutrient sensing RAS/Protein Kinase A (PKA) and TOR pathways [1–3] that promote ribosome production, translation, and proliferation while suppressing activators of the stress response [2, 4]. But upon exposure to severe stress, cells often down-regulate growth promoting functions while mediating myriad other changes, including in transcription, translation, and post-translational protein modification. Together, these rearrangements produce temporary delay of cell-cycle progression, altered metabolic fluxes, redistribution of the actin cytoskeleton, and other responses. Many of these processes are regulated by post-translational protein phosphorylation; but how cells coordinate many different processes with a single regulatory network during a systematic response remains unclear.

Many studies have characterized phospho-proteomic changes in mutant cells lacking stress-activated regulators. Downstream phosphorylation events requiring those regulators can be readily identified, but most are unlikely to be direct [5]. For example, in the well-studied response to osmotic shock, the HOG pathway is activated to coordinate osmo-induced transcriptome changes [6–8], translation regulation [9–11], cell cycle arrest [12–14], actin reorganization [15–17], and metabolic changes including those that produce intracellular osmolytes [18–20]. However, most of the phosphorylation sites related to these processes do not match the known specificity of Hog1 and are likely controlled by other downstream kinases [21]. Hog1-independent regulators are also activated during osmotic shock [17, 21–23], and other regulators remain to be identified. How these connect in a single regulatory network remains unknown.

Graph-based optimization algorithms can be used to extract a small, high-confidence subnetwork from a larger network of interactions, *e*.*g*. protein-protein interactions (PPI), to explain how phosphorylation signals propagate [24, 25]. Unlike kinase enrichment algorithms [26, 27], subnetwork inference places signaling regulators in a hierarchical pathway context. Subnetwork inference also offers advantages over *de novo* network inference [28, 29] when sample sizes are limited. Diverse optimization strategies have been used for pathway identification from a background PPI network, including methods based on source-target paths [30, 31], network flow [32], Steiner tree variants [33–36], probabilistic graphical models [37], and integer linear programs (ILPs) [38]. General subnetwork identification methods are flexible and adaptable to a wide variety of datasets and biological contexts. However, they lack the ability to explicitly model feedback loops [39], account for perturbations [37, 38, 40, 41], predict interaction directions [31, 37, 38], or prioritize the most likely phosphorylation regulators. We demonstrate these tradeoffs and the benefits of constructing a customized ILP that is tailored to signaling pathway inference with wild-type and regulator mutant phospho-proteomic data.

The current work builds upon our previous ILP method designed to capture the transcriptome-regulating network by integrating gene-fitness contributions to stress tolerance, wild-type and mutant transcriptomic responses, and phospho-proteomic changes triggered by the osmotic and ionic stress of sodium chloride (NaCl) treatment [25]. The resulting network enabled many new predictions about the NaCl transcriptomic response, by identifying new regulators and the regulatory connections between them. However, the inferred network included only 21% of proteins with significant phosphorylation changes during NaCl stress. This is reasonable, because most proteins with phosphorylation changes do not regulate the transcriptome but rather coordinate other aspects of the cellular response [42]. However, it indicates that regulation of other physiological aspects of the stress response beyond transcriptome changes is not captured by the previously published method.

Here, we devised a new approach specifically designed to infer the NaCl-activated signaling network that controls the phosphoproteome. Our approach first identifies submodules of likely co-regulated phospho-peptides that share similar phospho-motifs and mutant dependencies, and then implicates proteins from the PPI network that interact with many of those target peptides; this generates new edges that are added to the PPI network to connect potential regulators to downstream targets. The ILP then integrates data to infer a single signaling network, linking upstream regulators we interrogated to downstream phospho-peptide modules dependent on their activity. The method revealed exciting new insights into cellular coordination of disparate physiological responses to stress, several of which we validated through molecular approaches. In particular, the network illuminates cell coordination of cell cycle, metabolism, and growth control during acute stress and points to previously unknown connections between stress-activated and growth-regulating pathways.

## Results

We first profiled the phospho-proteome of wild-type and mutant cells before and after NaCl response. While other studies have interrogated osmo-responsive phospho-proteome changes [17, 21, 25, 43], for optimal network inference we restricted our analysis to data generated in our lab under the identical growth conditions (and used results of other studies as validation metrics of the approach). A full description of the phospho-proteomic data collection is found in the S1 Supporting Information Section 1. In summary, we used isobaric tagging and mass spectrometry to quantify 8,120 peptides, mapping to 2,049 proteins that were phosphorylated before and/or at 5, 15, or 30 min after treatment with 0.7M NaCl. We leveraged replicates at specific time points to identify 1,249 peptides from 618 proteins that showed reproducible phosphorylation changes (S1 Table) in wild-type cells responding to NaCl treatment (FDR < 0.05 or meeting our selection criteria, see Methods). These included 479 peptides (38%) with increased phosphorylation and 770 peptides (62%) with decreasing phosphorylation at some time after acute NaCl exposure.

Identifying phospho-peptides dependent on specific regulators enables the ILP method to connect those regulators to downstream phosphorylation targets. We therefore also profiled mutants lacking regulators implicated in our previous network, including the osmo-activated Hog1 kinase, the cAMP phosphodiesterase Pde2 that regulates PKA activity [44], and cell-cycle modulating Cdc14 phosphatase. We identified phospho-peptides with reproducible phosphorylation defects upon NaCl treatment (see Methods), implicating 211 defective phospho-peptides in the *hog1*Δ strain and 140 in the *pde2*Δ strain (S2 Table). To investigate Cdc14, we used the temperature-sensitive *cdc14-3* mutant paired with an identically treated, isogenic wild type to identify 161 phospho-peptides with in some cases subtle but reproducible phosphorylation defects (see Methods) (S2 Table); these were linked to budding, cell polarity and filament formation, GTPase signal transduction, and kinase activity (P < 1x10^-4^, hypergeometric test [45] (S3 Table), as expected if Cdc14 is inhibited.

### Phospho-proteome network inference

**Fig 1:**
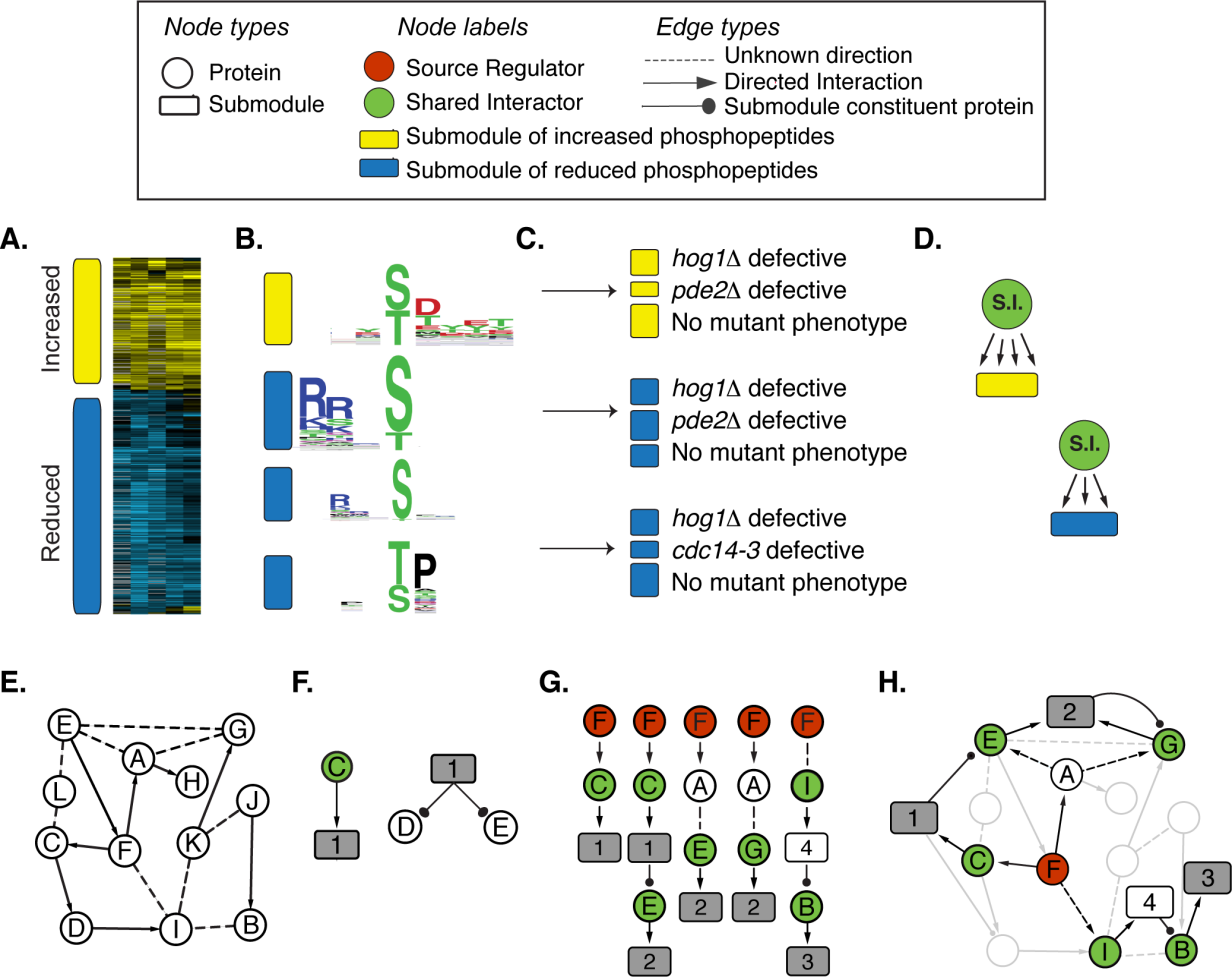
Overview of the inference method. The method consists of three main steps (see text for details). In the first step, stress-altered phospho-peptides are partitioned into submodules of peptides likely to be co-regulated. This is accomplished by **(A)** clustering phospho-peptides based on their pattern of phosphorylation change in wild type cells, then **(B)** further partitioning peptides into submodules if they share the same phospho-motif and **(C)** if they share the same defect in each of three interrogated mutant strains. Yellow and blue filled submodules are comprised of peptides with increased or decreased phosphorylation, respectively, in response to NaCl. **(D)** We then identify ‘Shared Interactors’ (SIs, green circles) as proteins that show more physical interaction with submodule constituent proteins than expected by chance–identified SIs are connected to each submodule with a new directional edge. **(E)** A background network of previously measured protein-protein (undirected dashed line) and kinase-substrate (directed arrow) interactions, represented here with Proteins A–L, is augmented by **(F)** adding SI-submodule units as well as outgoing edges (ball and stick) between each submodule and its constituent proteins whose phospho-peptides belong to the submodule. **(G)** The ILP method then enumerates all paths of a given length from each source regulator (red) to its dependent submodules (grey boxes), traversing through SIs (green) and other proteins in the augmented PPI background network. In this cartoon, submodules 1, 2, and 3 consist of phospho-peptides whose salt responsiveness depends on interrogated source regulator Protein F. Submodules without a source dependency (white boxes) can be incorporated as pathway intermediates. **(H)** The ILP connects the units using a multi-stage objective function to reveal the subnetwork inferred to regulate phosphoproteome changes.

We adapted our prior ILP-based approach to integrate the wild type and mutant phospho-proteomic changes into a single regulatory network, by linking the three interrogated regulators to their downstream targets defined in the mutant phospho-proteomic analysis. A key advance of the new approach is that we add edges to the background network between implicated regulators and groups of potential targets. The method works through three main steps (Fig 1, complete details in S1 Supporting Information Section 2):

a) Partition phospho-peptides into potentially co-regulated modules. Many kinases recognize specific phosphorylation motifs and act on suites of targets that harbor those sites [46]. Thus, we identified groups of phospho-peptides that share specific phenotypes, and therefore may be co-regulated. First, we grouped peptides into those with increased or decreased phosphorylation after NaCl treatment (Fig 1A). Next, for each group we partitioned peptides based on shared phosphorylation motifs, using the program *motif-X* [47, 48] (Fig 1B). This implicated 17 **modules** of peptides (capturing 71% of NaCl-responsive phospho-peptides) that showed similar directionality in phosphorylation change and shared phosphorylation motifs. Finally, we partitioned each module of peptides based on their dependencies on each of the three interrogated regulators (see Methods). This generated 76 **submodules** of peptides (Fig 1C), including 16 submodules (capturing 159 peptides) that were dependent on Hog1, 19 submodules (100 peptides) dependent on Pde2, 24 submodules (110 peptides) affected by Cdc14, and 17 submodules (563 peptides) with no detectible dependency on any of the three regulators (S4 Table). We note that some legitimate targets of the three regulators are likely incorporated in submodules with no nominal mutant phenotype, if the mutant effect fell below our fold-change criteria.

b) Implicate potential regulators linked to each submodule. Under the assumption that co-regulated peptides should interact with the same responsible regulator, we leveraged the PPI network to implicate proteins that display more interactions with submodule proteins than expected by chance (FDR < 0.05, hypergeometric test, see Methods) (Fig 1D). We refer to these as **shared interactors** (SIs). A key advantage of this strategy is that it can overcome missing interactions in the published background network, since the SI need not interact with every protein in the submodule (see below). This is an important advance, because PPIs suffer from many false negative (*i*.*e*. missing) interactions. Using this approach, we identified a total of 472 SIs for 54 of the 76 submodules (S5 Table). The SIs included 71 kinases, 6 phosphatases, and many proteins of other functional classes—we note that many SIs may not be direct regulators, but instead represent other types of protein interactors (*e*.*g*. proteins in complex with submodule proteins). SI kinases whose known specificity matched the submodule phosphorylation motif ([49], Kullback-Leibler Divergence, FDR < 0.2%, see Methods) were elevated in confidence as the direct regulator of the submodule peptides (although not prioritized by the ILP since many kinases show relaxed specificity, see Discussion).

c) Link proteins into a network using ILP. We aimed to connect the SI-submodule units into a signaling network by traversing the augmented background PPI network (Fig 1E). The ILP connects interrogated regulators (Hog1, Pde2, Cdc14, referred to as **sources**) to downstream submodules whose peptides require that regulator for phosphorylation. To do this, we first updated the background network to include edges from each SI to its associated submodules and edges from each submodule to **constituent** proteins whose peptides are included in the submodule (Fig 1F). We then enumerated all possible directed paths of length 3 (discounting submodule-constituent edges) from each source to its dependent submodules (Fig 1G). The ILP then identifies the subnetwork of paths that connect the three source regulators to all downstream submodules, minimizing the number of nodes but maximizing the inclusion of SIs. Many related networks may score equally well; therefore, the output is an ensemble of high-scoring networks with directed, linear paths between sources and submodules (Fig 1H). Because we had few source regulators, we applied a strategy for increasing network diversity within candidate paths [50] by repeating the network inference 1,000 times and each time holding out 5% of proteins from the previous solution. This approach resulted in a richer consensus network compared to our previous approach [25], doubling the number of non-SI proteins included in the network and quadrupling the inclusion of non-SIs involved in osmotic stress (see Methods). The resulting ensemble was collapsed into a consensus network, with node and edge confidence values taken as the fraction of ensemble networks in which they were identified. As a final step, we added back submodules that were not included in the consensus but whose SIs were represented. This allowed inclusion of submodules with no detectable mutant dependency, enabling predictions beyond the three source regulators.

**Fig 2:**
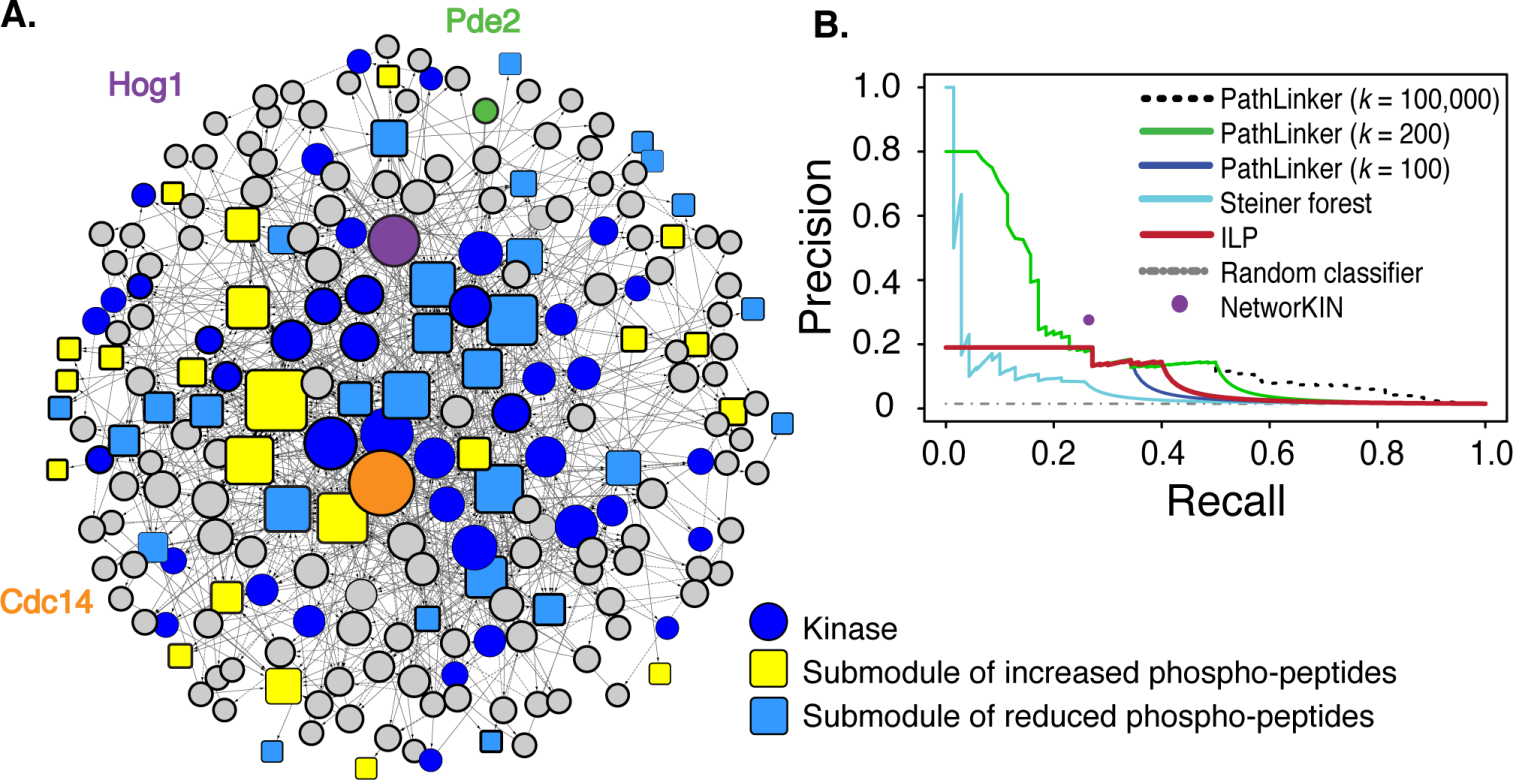
Inferred NaCl-activated phosphorylation signaling network. **(A)** Consensus network at 75% confidence where node size represents degree. Pde2, Hog1, and Cdc14 sources are denoted with green, purple, and orange circles, respectively. Rectangular submodules are colored yellow or blue if their phospho-peptides showed increasing or decreasing phosphorylation upon NaCl treatment. (**B)** Precision-recall curves were calculated using a true positive list, excluding submodules and sources (see Methods and S1 Supporting Information Section 3 for evaluation details). *Precision* is the percentage of network proteins that are true positives, while *Recall* is the percentage of true positives retrieved.

After supplementing with no-phenotype submodules not included by inference (see Methods), the resultant consensus network (at 75% confidence) contained 167 proteins in regulator paths and 51 submodules (encompassing 832 of 1249 phospho-peptides), with 844 edges between them (Fig 2A). The network included 75%, 36.8%, and 62.5% of submodules dependent on Hog1, Pde2, and Cdc14, respectively. The network also predicts the directionality of information flow, suggesting upstream regulatory proteins and downstream targets.

### Computational validation provides strong support for the inferred subnetwork

We assessed the predictive accuracy of the network in several ways. First, the network captured many known regulators of the NaCl response, including proteins in the HOG, RAS/PKA, TOR1, CK2, and Snf1/AMPK pathways. Second, we found that the inferred network was enriched for expected proteins, including kinases (*P* = 3.5x10^-36^, hypergeometric test), proteins that interact with Hog1 or in the HOG pathway (*P* = 8.4x10^-26^) [51–53] and proteins annotated with ‘osmotic’ or ‘stress’ response (*P* = 1.9x10^-14^ [54, 55]). Third, we compared to functional data, leveraging a screen that identified genes important for stress survival after NaCl treatment [56]. We found that the network was enriched for proteins required for NaCl-dependent acquisition of future stress tolerance (*P* = 1x10^-3^).

Some of the method performance could be influenced by the structure of the PPI background network, independent of the biology [25]. We tested this by applying the method to scrambled PPI data that maintained the network degree structure ([57], see Methods). The networks generated from scrambled data fared significantly worse than the network inferred from real data, based on precision and recall of true positive regulators and connection of SIs to targets submodules (see S1 Fig and Table 1 below). Thus, while there is some influence of the PPI network structure, the ILP-inferred ensemble network captures biology relevant to yeast signaling.

**Table 1:** Comparison of the ILP, prize-collecting Steiner forest (PCSF), and PathLinker (PL) network inference methods.

Inference Method	Protein	SI	Total Submodule Nodes*	NP-Submodule Nodes**	Edges	Precision
	Nodes	Nodes				
ILP	167	143	46	12	844	0.143
ILP–scrambled	124^a^	39^b^	33^a^	5^a^	283^a^	0.065^a^
PCSF	215	91	37	0	511	0.083
PL *k* = 100	161	102	32	3	455	0.149
PL *k* = 200	248	138	42	7	799	0.141
PL	2,165	419	54	17	20,897	0.029
*k* = 100,000						

*Total Submodule Nodes lists those with and without mutant phenotypes captured by the inference.

**NP Submodule Nodes lists the number of no-phenotype submodules captured by inference.

^a^ Average numbers across 5 ILP networks inferred from scrambled background data.

^b^ The number of those SIs captured in the real ILP network.

We next compared our method to three other established procedures (see S1 Supporting Information Section 3 for full details). NetworKIN is a commonly used method that matches kinases to potential protein targets, if the phospho-motif matches the known kinase specificity [26]; however, it does not infer a broader network and can only match kinases if their specificity is known. Nonetheless, our approach identified significantly more HOG network components and many more kinases (S1 Supporting Information Section 3 and Fig 2B). We also compared to a prize-collecting Steiner forest method (PCSF), which awards prizes for including proteins known to be important and penalizes edges to control the subnetwork size [34], and PathLinker, which uses a shortest path approach to prioritize paths in a weighted background network [30]. Unlike our ILP, neither the Steiner forest method nor PathLinker can specify specific targets for multiple source regulators. Therefore, we ran the methods separately for each source and combined the pathways (see Methods).

We compared methods with several metrics, including precision and recall in recovering known true-positive osmotic regulators (see Methods), the fraction of target submodules incorporated into the network, and known biological connections captured. Our method outperformed the Steiner forest method by all metrics, with greater area under the precision-recall curve (AUC) and more target submodules incorporated (Fig 2B, Table 1, S1 Supporting Information Section 3). PathLinker returns ranked source-target paths, which we used to order nodes for our precision-recall analysis (see Methods). We compared performance to the complete list of ranked nodes for *k* = 100,000 paths as well as nodes identified in the top *k* = 100 and *k* = 200 highest scoring paths, which produced networks of similar size to the ILP (Table 1, see Methods). Although the AUC was higher for PathLinker networks, this was primarily due to fine-grained resolution of high-confidence node ranking (leading to high precision for small networks plotted on the left of the curve). The ILP returned a group of 100 nodes with maximal confidence, producing a recall of 0.27 and precision of 0.19. PathLinker reached a recall of 0.27 with a similarly sized network of 104 nodes and precision of 0.18, nearly the same as the ILP. Beyond that point, both methods rank nodes with comparable accuracy, though PathLinker can rank many more nodes if the user does not seek a parsimonious subnetwork and uses a large *k*.

However, PathLinker performed worse on other metrics, leading us to prefer the ILP for biological interpretation and prioritization of validation experiments. The comparably sized PathLinker *k* = 100 and *k* = 200 networks captured fewer source-regulated target submodules and fewer SIs (the latter by design, because the ILP prioritizes them). PathLinker networks also captured substantially fewer no-phenotype submodules that are added back to the network in the last step, preventing several insights afforded by the ILP network (S1 Supporting Information Section 3). Finally, the PathLinker network structure did not conform to the typical signaling pathway topology but instead produced extensive looping for some nodes (S2 Fig, S1 Supporting Information Section 3). Thus, the ILP outperformed NetworKIN, the Steiner forest, and PathLinker for biological interpretation of signaling responses.

### The NaCl-responsive phospho-proteome network captures different functional categories than the previously inferred transcriptome-regulating network

**Fig 3:**
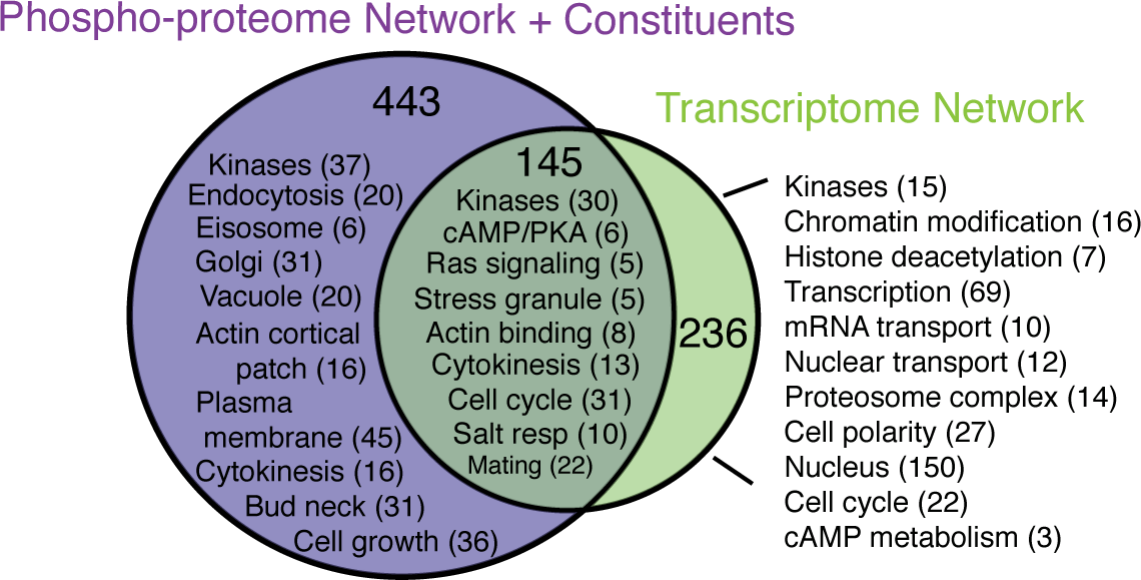
The NaCl-activated networks regulating the phospho-proteome and transcriptome capture unique functional categories. A summary of enriched GO categories (P<1x10^-4^ [45]) for proteins shared and unique to each network. Complete GO enrichments are in S6 Table.

A main motivation was to complement our previously inferred transcriptome-regulating network, so as to more broadly capture cellular signaling and physiological coordination during stress. We expected that, if the inferences are working properly, the two networks should capture proteins involved in different processes, and indeed this is the case (Fig 3). The previously inferred transcriptome-regulating network was heavily enriched for proteins involved in transcription, mRNA transport, chromatin modification, and those localized to the nucleus, among other functions including proteasome degradation (*P* < 1x10^-4^, hypergeometric test [45], S6 Table). None of these functions was enriched among the 443 proteins uniquely included (either in regulatory paths or as submodule constituents) in the phospho-proteomic network. Instead, this group was uniquely enriched for proteins involved in endocytosis or found within the eisosome, Golgi apparatus, actin cortical patch, or plasma or vacuole membranes. Annotations related to cell cycle progression, actin cytoskeleton and kinases were enriched among proteins unique to both networks. Beyond these, 145 proteins were identified in both networks and these were enriched for kinases, known osmotic and stress-response regulators, and proteins involved in RAS and PKA signaling, as well as regulators of cell cycle progression/cytokinesis and actin cytoskeleton [45] (*P* < 1x10^-4^) (Fig 3). We propose that many of these represent master regulators coordinating transcriptome changes with other physiological responses to NaCl stress. Indeed, many upstream regulators–including HOG and PKA pathway components–were included in both networks.

### Coordination of cell-cycle control during osmotic shock

Bolstered by the results above, we set out to interrogate the inferred phospho-proteome regulating network, for biological predictions and to provide additional support for the approach. We were interested in leveraging several advantages of our approach compared to other established methods. One key advance is that the method can predict phosphorylation targets by nature of SIs, even when no physical interaction between a predicted regulator and its target has yet to be measured in published datasets. This is important, since interaction datasets are missing many interactions [58], especially those that may be stress specific [59]. Another advantage is that our method can capture feedback loops, which are common in cellular signaling. Feedback is captured when a constituent of a submodule is connected back to that submodule, either as a SI or in a path leading back to that submodule. These provide useful benefits that can expand our understanding of the signaling network.

**Fig 4:**
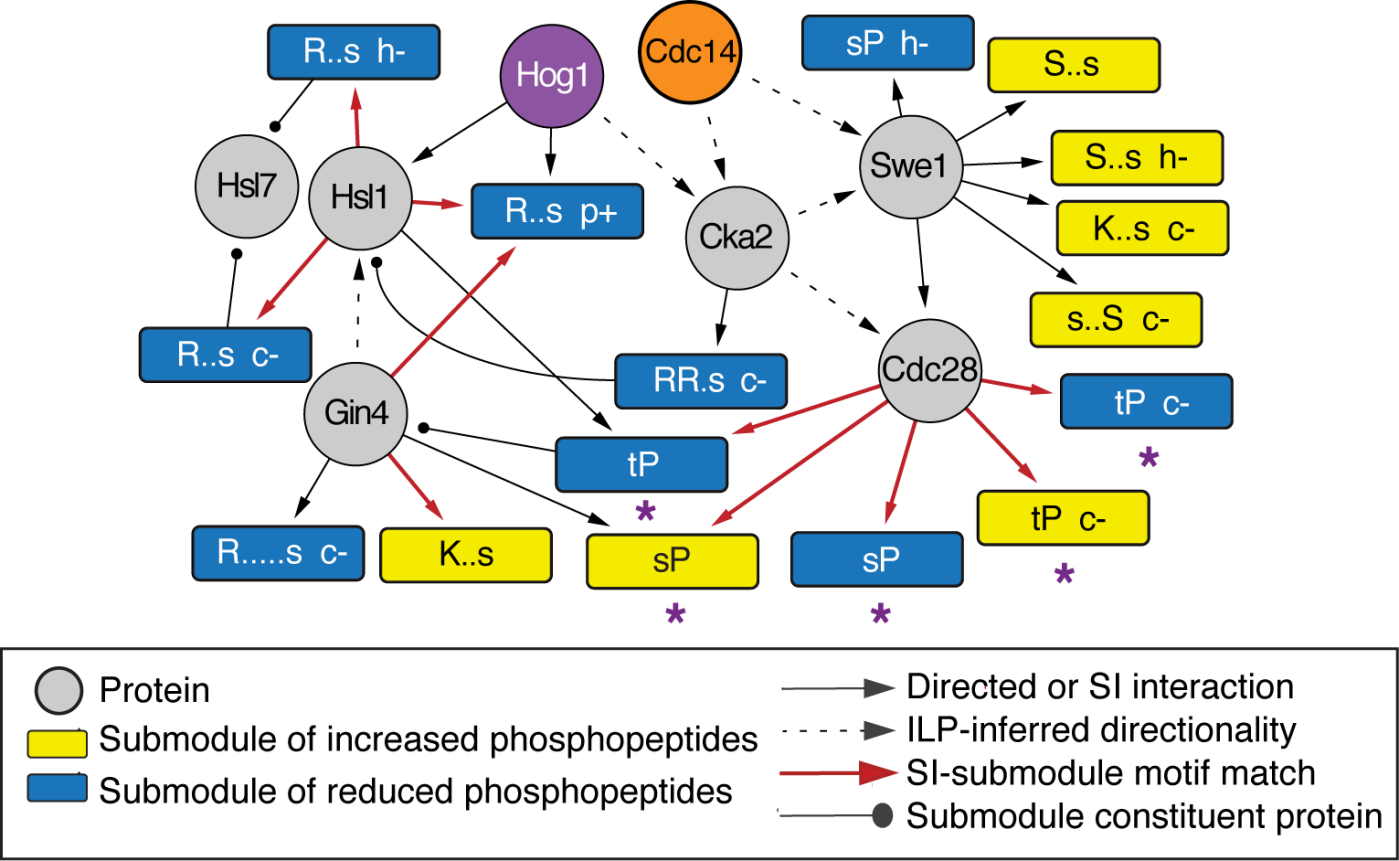
Subnetwork related to cell cycle control. A manually chosen region of the network capturing known regulation between Hog1 and Hsl1 (see text), other regulators connected to Hog1 or Hsl1, and all submodules connected to those regulators. Submodules are annotated by the phospho-motif and mutant phenotype if peptide changes were defective (-) or amplified (+) in the *hog1*Δ (‘h’), *pde2*Δ (‘p’), or *cdc14-3* (‘c’) mutants, and colored according to the key. Solid arrows represent directed SI-submodule edges or known directional interactions, dashed arrows represent directionality inferred by the ILP, and ball-and-stick edges indicate protein constituents of the submodule from which the line emits. Red arrows indicate a motif match between the known SI kinase specificity and the target submodule (FDR < 0.2). Asterisks denote submodules containing known Cdc28 target proteins, as curated in Chasman *et al* [52, 53], or phospho-peptides with defective phosphorylation in a strain in which Cdc28 was chemically inhibited [5, 61].

An example of these benefits is highlighted by the connections between the HOG and cell cycle pathways. Hog1 is known to mediate cell-cycle delay at several phases after osmotic shock. Arrest at G1 is triggered in part by Hog1 phosphorylation of the Cdc28 inhibitor Sic1 [13]. Arrest at G2 is coordinated by a cascade of events, when Hog1-dependent phosphorylation of Hsl1 inhibits its kinase activity, resulting in decreased phosphorylation and thus delocalization of its target Hsl7; this in turn enables accumulation of the Swe1 regulator that phosphorylates and inhibits Cdc28 [12]. Our inferred network captured many of these regulators and information flow between them (Fig 4). For example, the network correctly predicted that Hog1 directly phosphorylates Hsl1 and that Hsl1 is down regulated during NaCl treatment, since all of its connected submodules show decreased phosphorylation. These submodules include Hsl1’s known target, Hsl7, which shows Hog1- and Cdc14-dependent phosphorylation decrease on Ser-718. While Cdc14 is not a known regulator, it physically interacts with Hsl7 [60], supporting a direct regulatory connection.

The network also made novel predictions for cell cycle control during NaCl stress. Cdc28 was connected to several submodules whose phospho-motifs match the known Cdc28 specificity, and these spanned 81 phospho-sites with increased phosphorylation and 71 phospho-sites with decreased phosphorylation. Of the combined constituent proteins of all of these submodules, 24% are known Cdc28 targets [52, 53]. Our method predicts that other constituents may represent novel targets. To test this, we compared to two recent phospho-proteomic studies that inhibited *cdc28* analog-sensitive mutants under various conditions [5, 61]. Indeed, the group of predicted Cdc28 target peptides was heavily enriched for sites whose phosphorylation is affected by Cdc28 inhibition (*P* = 5.6 x 10^−15^, hypergeometric test). Only a quarter of these proteins have a known interaction with Cdc28 [52, 53]. Thus, our method elevates the remaining proteins as potential direct targets of Cdc28. Several of these submodules were dependent on the Cdc14 phosphatase, which is known to modulate Cdc28 activity toward different targets [60, 62, 63]. Taken together, these results strongly predict that many of these phospho-peptides are novel direct targets of Cdc28.

### Rck2 is key regulator in the NaCl-activated signaling network

**Fig 5:**
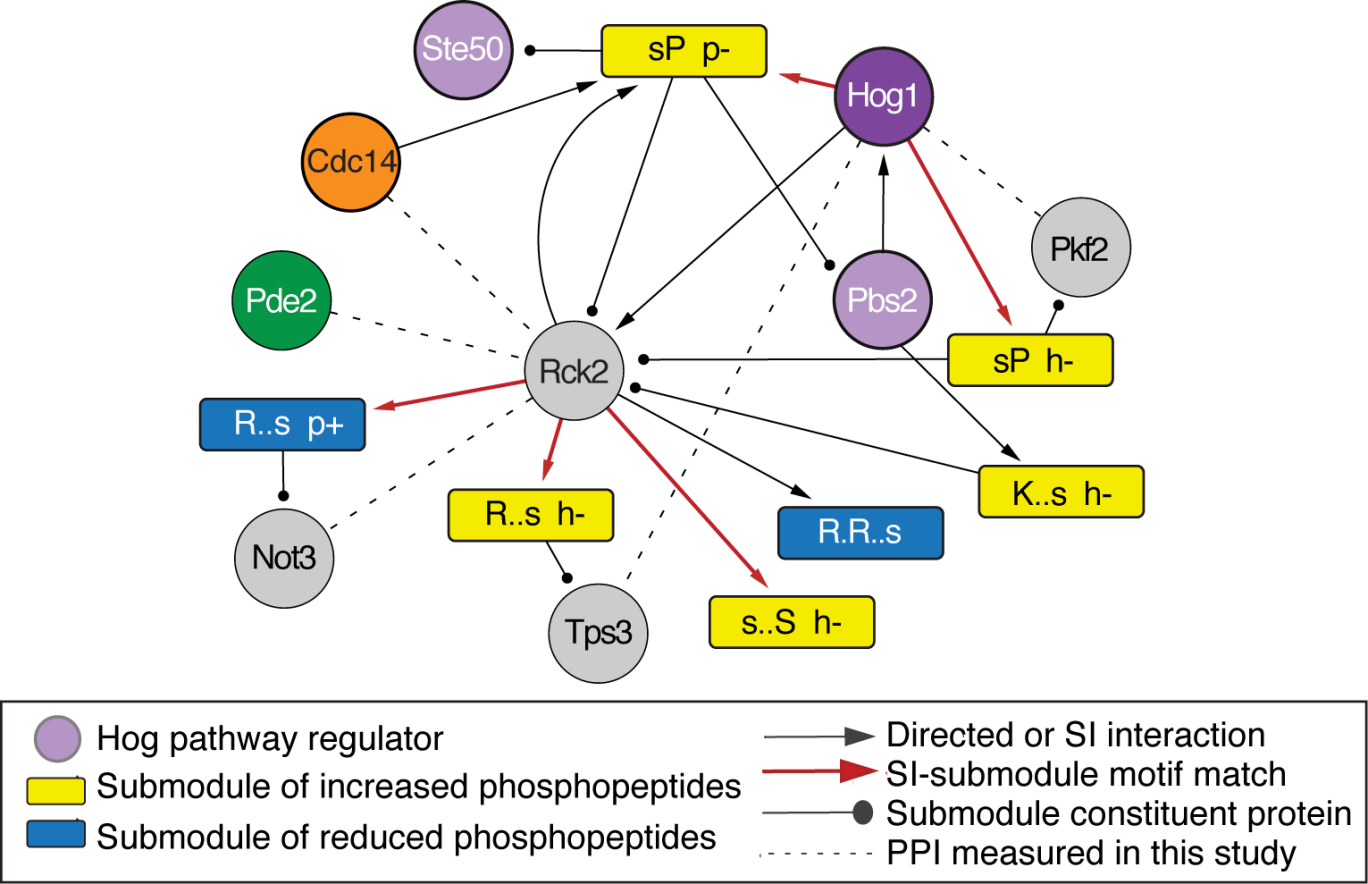
Rck2 is a hub in the osmotic stress-signaling network. A manually chosen section of the network capturing source regulators, Hog pathway components, and Rck2 shown, as described in Fig 4 and the key. The figure also shows three constituent proteins that are predicted targets of our method and whose physical interaction with source regulators we validated by co-immunoprecipitation (dashed lines).

In response to high osmolarity, Hog1 is known to phosphorylate and activate the kinase Rck2, which subsequently phosphorylates targets to decrease translation elongation [11, 64]. We capture a path from Hog1 to a submodule containing Rck2, and then from Rck2 to several submodules whose phospho-motif matches Rck2’s preference and whose phosphorylation is dependent on Hog1 (Fig 5). Only nine percent of the proteins within these submodules are known Rck2 targets [52, 65] leaving the remaining proteins and phospho-sites as novel predictions of our method. We compared these predictions to a recent phospho-proteomics study of an *RCK2* mutant subjected to osmotic stress [21]. Strikingly, phosphorylation of 44% of the novel predicted phospho-sites are dependent on Rck2 during osmotic stress (*P* = 2.7x10^-14^, hypergeometric test). One of the constituents is a phospho-peptide mapping to Tps3, a regulatory subunit of the trehalose synthase [66]. While this site on Tps3 Ser-974 has not been linked to Rck2, Romanov *et al* demonstrated that Rck2 likely phosphorylates several other Tps3 sites [21]. Our results propose that Rck2 also phosphorylates Tps3 on Serine 974.

This region of the inferred network captured several other interesting connections. One of the Hog1-connected submodules included Ste50, an upstream regulator known to be phosphorylated by Hog1 as part of a negative-feedback mechanism [67]. Indeed, the submodule captured the known Hog1 target site on Ste50, Ser-202 [67]. Interestingly, NaCl-responsive phosphorylation of the Ste50/Rck2-containing submodule was dependent on Pde2, and in turn one of Rck2’s connected submodules showed a Pde2-dependent phosphorylation change upon NaCl treatment. These results raised the possibility that Rck2 may also be regulated by Pde2 during NaCl stress. Cdc14 was also identified as an SI to the Rck2-containing submodule, although there was no detectible defect in Rck2 phosphorylation in the Cdc14 mutant.

To test these predictions, we immunoprecipitated GFP-tagged Pde2, Cdc14, Rck2, and Hog1 before and 10 min after salt stress and used mass spectrometry to identify other recovered proteins (S7 Table). Co-immunoprecipitations (co-IP) validated several of the predicted interactions: IP of both Cdc14 and Pde2 recovered Rck2, whereas IP of Rck2 pulled down Pde2 (albeit just below the threshold used to call co-IPs). Rck2 pull down also recovered Not3, a member of the CCR-NOT complex that was predicted as a novel Rck2 target. That Pde2 is required for Rck2 phosphorylation and predicted downstream Rck2 effects, coupled with the physical interaction between Pde2 and Rck2, strongly suggests that Pde2 is important for Rck2 regulation (see Discussion). IP of Hog1 validated another prediction by recovering the glycolytic enzyme phosphofructokinase Pfk2, which was not previously known to interact with Hog1 but was included in a submodule to which Hog1 was connected and showed Hog1-dependent phosphorylation upon NaCl stress (Fig 5). Together, these results confirm that the ILP network inference can make powerful predictions about regulatory connections.

### New connections and extensive feedback predicted in the protein kinase a network

A central player in the osmotic shock response is the PKA pathway, which promotes growth-related processes under optimal conditions but is suppressed to enable defense strategies [2, 68]. How PKA signaling is reduced during stress is not clear. A major portion of our inferred network captured responses linked to PKA. PKA subunits Tpk1, 2, and/or 3 were identified as SIs for 19 submodules, including 6 whose phosphorylation motifs matched the known PKA consensus (R/K-R/K-x-S/T) and an additional 5 matching the lower affinity motif (R-x-x-S) [49, 65, 69]. All of these submodules showed decreased phosphorylation in response to NaCl treatment consistent with reduced PKA activity. The constituent proteins in these submodules are enriched for known PKA targets [52, 53] (*P* = 1.1x10^-5^, hypergeometric test); additional peptides were linked to PKA either through genetic [70] or physical interactions with PKA subunits [71] or defective phosphorylation in PKA catalytic mutants [72].

Interrogating the network revealed new links between PKA signaling and physiology. Collectively, the constituents of PKA-connected submodules with reduced phosphorylation upon NaCl were enriched for proteins involved in budding, cell polarity and filament formation, GTPase- and cAMP-mediated signal transduction, stress response, cell wall structure, and included many kinases (*P* < 1x10^-4^) (S8 Table). Fourteen transcription factors were also featured in these submodules (see more below), including stress-responsive factors such as Msn2, Crz1, Sko1, and Dot6 that are inhibited by direct PKA-mediated phosphorylation [73–76]. Of the 17 kinases connected to PKA, over two-thirds are not known to be PKA targets but could represent novel downstream pathways mediated by PKA signaling.

**Fig 6:**
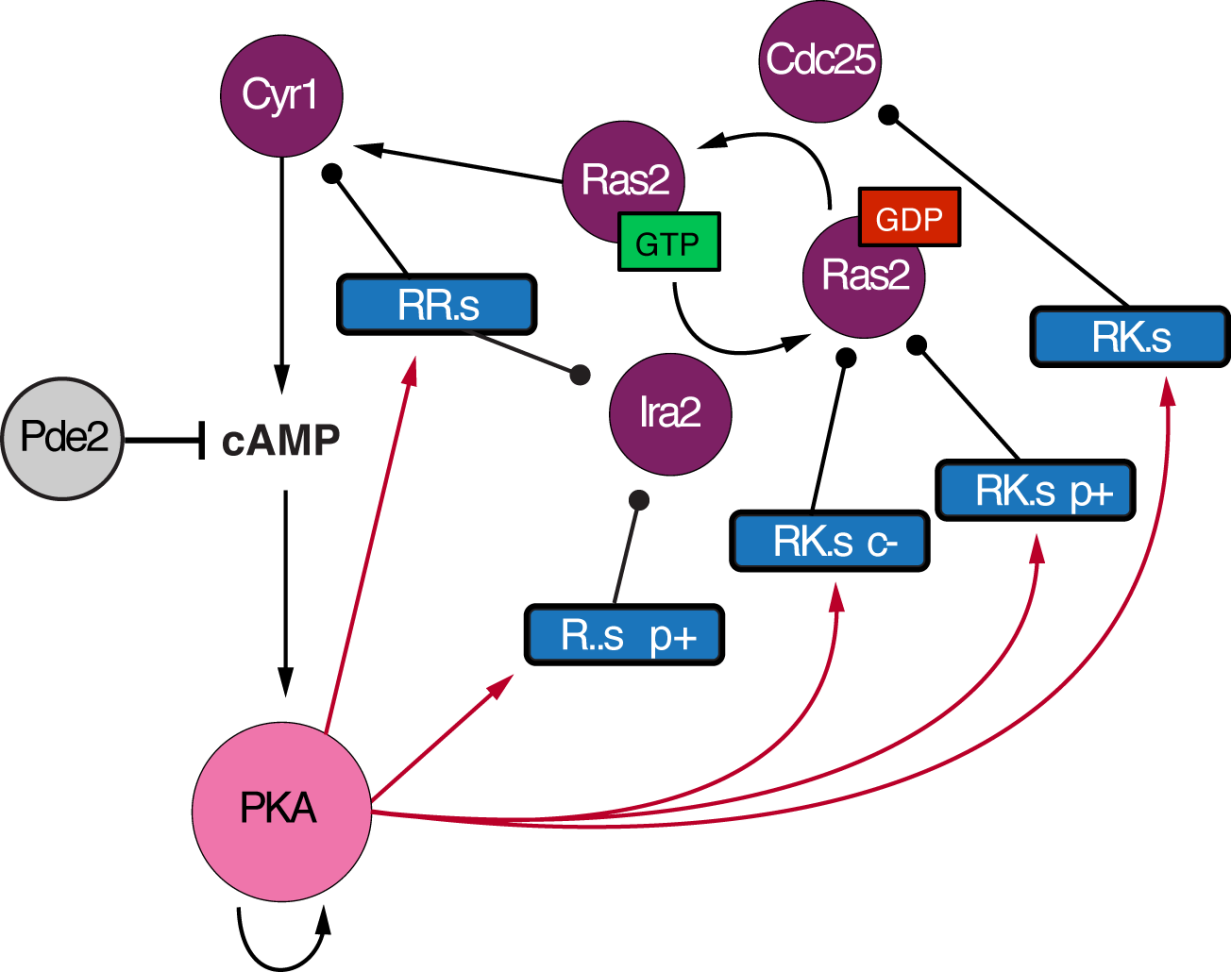
Inferred feedback in the PKA pathway. Shown are all submodules connected to at least one PKA catalytic subunit (‘PKA’) and containing a known regulator in the PKA pathway. Pink and purple filled circles represent the PKA catalytic subunits and members of the PKA pathway, respectively. Submodules are colored according to the key and annotated by the phospho-motif and mutant phenotype if peptide changes were defective (-) or amplified (+) in the *pde2*Δ (‘p’), or *cdc14-3* (‘c’) mutants. Inhibition of PKA by Pde2-dependent cAMP decay is also represented. Red arrows indicate a motif match between at least one PKA kinase subunit and the target submodule (FDR < 0.2). Ras2 is shown in its active (bound to GTP) and inactive states (bound to GDP). The predicted PKA auto-phosphorylation is on the Tpk3 subunit.

Remarkably, many of the PKA-connected submodules included proteins in the RAS/PKA signaling pathway itself (including Cdc25, Ras2, Cyr1, Ira2, and Tpk3, *P* = 4.8x10^-3^), suggesting extensive feedback control in the PKA pathway (Fig 6). Phosphorylation of several of the captured sites, including Cdc25 Ser-135 and Ras2 Ser-214, has been suggested to play a role in PKA feedback regulation [77–80]. The network also captured phospho-sites of unknown function on the adenylate cyclase Cyr1 (S60), catalytic subunit Tpk3 (S15), and the negative regulator of RAS, Ira2 (S433 and S1018). Phosphorylation of Ira2 (S1018) was previously shown to be decreased in a *TPK3* mutant [72], supporting our supposition of a direct PKA effect. Future studies will be required to dissect the specific effects, but these results suggest significant feedback signaling in the PKA network, which to our knowledge has not been captured previously in inferred regulatory networks.

### Pde2 interacts with many transcription factors whose phosphorylation is regulated by PKA and Rck2

**Fig 7:**
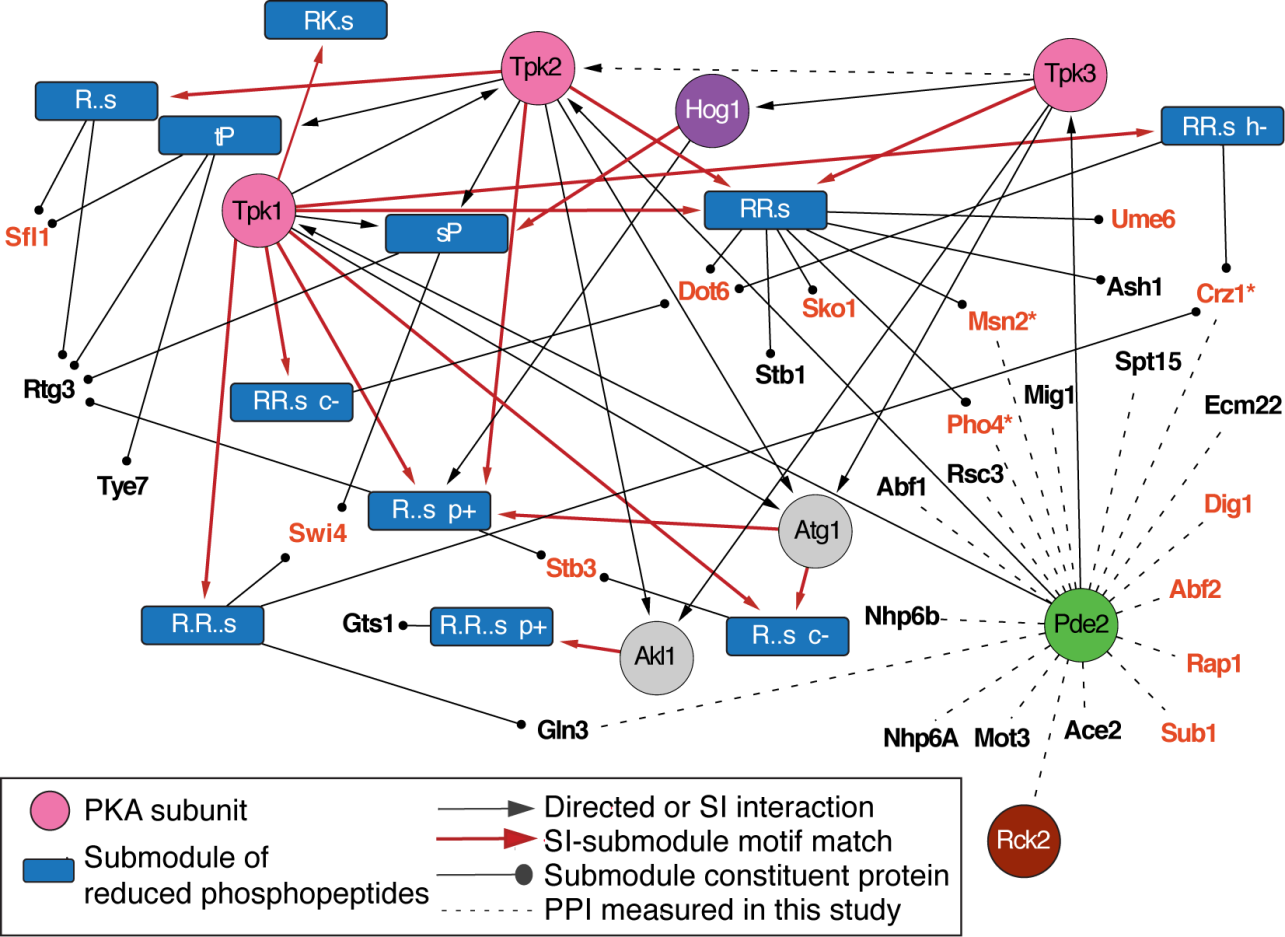
Pde2 interacts with stress-regulated transcription factors. Shown are 10 phospho-repressed submodules (blue rectangles) downstream of at least one PKA subunit and containing at least one transcription factor, as described in Fig 4. Dashed lines without arrows denote Pde2 protein interactions identified by co-IP. Bolded red text denotes factors that are either known or predicted PKA targets [52, 53] or reside in pathways directly regulated by PKA [83–85, 87].

PKA was connected to many downstream transcription factors, some previously known to be PKA controlled. Several of these, including the general stress factors Msn2/4, Sko1, and Dot6 and calcium-responsive Crz1, lie at the interface of PKA and stress-activated pathways [73, 74, 81, 82]. We noticed that deletion of *PDE2* affected the phosphorylation of many peptides, but relatively few of these were predicted to be directly connected to PKA. Although there is a second phosphodiesterase in yeast (Pde1) that could provide redundancy in PKA regulation, the lack of *pde2*Δ effect on PKA-linked phosphorylation was surprising, since deletion of *PDE2* produces a major defect in salt-responsive transcriptomic changes [25] and causes corresponding defects in acquired stress tolerance after NaCl treatment [56]. But an interesting result emerged from IP of Pde2: nearly a quarter of co-IP’d proteins (17 of 73) were transcription factors, which is much higher than predicted by chance (*P* = 2.6x10^-10^) (Fig 7 and S7 Table). Three of these (Msn2, Crz1, and Pho4) are known or predicted PKA targets [65, 73, 74, 81] and another four (Abf2, Rap1, Dig1, Sub1) are part of pathways regulated by PKA [83–87]. Pde2 is critical for normal induction of Msn2/4 targets [25]. This result supports a new model that Pde2 may directly interact with transcription factors to locally regulate cAMP levels (see Discussion).

How Pde2 is activated during stress is not known. Our aforementioned finding that Pde2 physically interacts with Rck2 raised the possibility that Rck2 may also influence Pde2 and/or PKA activity. If true, we would expect the *rck2*Δ mutant to have a defect in the salt-dependent decrease in phosphorylation of PKA targets predicted in our network. To investigate, we analyzed the phospho-proteomic response of an *rck2*Δ mutant responding to high NaCl [21]. Strikingly, 35% (118 of 335) of peptides with ≥2-fold higher phosphorylation in the NaCl-treated *rck2*Δ mutant versus wild type harbored the PKA motif (*P* = 4.4x10^-48^). Furthermore, this effect pertained to nearly a quarter of the constituents of PKA-associated, phospho-repressed submodules in our study, more that expected by chance (*P* = 1.8x10^-11^). Many of the affected proteins are known PKA targets, including stress-responsive transcription factors Crz1, Sko1, Yap4, Sfl1, Msn2/4, Maf1, Dot6, and Ifh1 and Rtg1. Other predicted PKA targets were also affected, including Bcy1, Cdc25, Pfk26, and Nth1 that contribute to PKA signaling, growth, or metabolism. Phosphorylation of several of these sites promotes cell growth, including Serine 178 on Pol III repressor Maf1 that alleviates Maf1-dependent repression of rDNA transcription [88]. Taken together, these results suggest that Rck2 plays a role in antagonizing PKA-mediated growth promotion during NaCl stress (see Discussion).

## Discussion

Here we present a novel computational approach to infer stress-activated signaling networks as well as discover new biology in the yeast salt-stress response. The results not only validate the approach but also highlight the novel insights that can be afforded by network inference.

Our computational method offers several contributions. Connecting SIs to groups of likely co-regulated phospho-peptides enables the prediction of novel protein interactions that are currently missing from the protein interaction network. While these remain predictions until proven by other methods, they present new hypotheses for subsequent direct testing. In spite of the high false-negative rate of co-IP analysis via mass spectrometry [89–91], we successfully validated several new predicted interactions (Fig 5). This included interactions between Rck2, Cdc14, Hog1, and several predicted targets (S7 Table). Another advantage is that SI-submodule relationships predict not only the targeted protein but also the specific phospho-site modified by that regulator. We showed that predicted target sites were indeed defective in cells lacking functional Cdc28, Rck2, or PKA. Our capacity to predict both the upstream regulator and the targeted site(s) presents an exciting opportunity to test the specific effects of those sites, through phospho-mimicking mutations [92]. Finally, the ability of our approach to predict signaling feedback will be particularly useful in dissecting how cellular signaling is amplified, attenuated, and augmented by pathway activation.

We augmented our previously published ILP approach to infer a signaling network that incorporates SI-submodule and submodule-constituent edges. The ILP multi-stage objective function provides flexibility in the inference, allowing the integration of mutant phospho-proteomic data and domain-specific knowledge including SI-submodule connections. The ILP approach outperformed both NetworKIN and the Steiner forest method in capturing known positive interactions (Fig 2B), and surpassed all three methods in incorporating known osmo-regulators, SIs and submodules that are targets of the source regulators (Table 1).

Despite these advances, there are several limitations of our approach as currently implemented. One challenge is in how peptides are partitioned into likely co-regulated sites. On the one hand, co-regulated targets may be inappropriately split into multiple submodules due to overfitting. For example, PKA can phosphorylate both RKxS and RRxS sequences [65], yet *motif-X* partitions peptides containing these motifs into separate modules. Such overfitting may hinder the subsequent identification of SIs due to small numbers of constituents in each submodule. On the other hand, sites recognized by different kinases can in theory be incorporated into the same submodules, if those kinases share the same specificity and mutant dependencies. Proline-directed Hog1 and Cdc28 both recognize SP motifs while PKA and Sch9 regulate basophilic sequences [49]. This is a key consideration when formulating hypotheses for subsequent testing. Finally, some kinases do not have strong sequence recognition motifs [49], and consequently their targets may not share a phospho-motif. Our method primarily uses phospho-motifs to partition peptides into likely co-regulated groups; while we visually represent edges where known SI kinase specificity matches the submodule motif, motif-match edges were not prioritized in the ILP because of the potential for kinases to recognize other sequences. We note that other methods could be used to identify co-regulated peptide groups, including shared docking motifs [93].

Nonetheless, the inferred network proved powerful in providing insights into stress biology, including the interplay between stress defense and growth-promoting pathways. PKA regulators were prominently featured in the network, suggesting myriad new PKA targets involved in cytoskeletal rearrangements, growth control, feedback signaling and stress-dependent transcription. How PKA is down-regulated during the NaCl response remains unclear. Deletion of *PDE2*, one of the two cAMP phosphodiesterases in yeast, did not produce a major defect in predicted PKA-dependent phosphorylation events despite a major defect in the NaCl-responsive transcriptomic changes [25]. In mammalian systems, phosphodiesterases have been implicated in creating micro-environments of low cAMP for local PKA control [94–96], reviewed in [97, 98]. One possibility is that Pde2 locally regulates cAMP surrounding transcription factors, *e*.*g*. in the nucleus or on the target-gene promoters, rendering a major defect in gene expression but little apparent effect on bulk cellular phosphorylation profiles. Future experiments will be required to test this model. Our analysis also suggests that Rck2 plays an important role in PKA regulation. Data from Romanov *et al*. show that the *rck2*Δ mutant treated with NaCl shows significantly higher phosphorylation of many PKA targets compared to a NaCl-treated wild type [21]. Interestingly, *hog1*Δ mutants (in [21] and here) do not produce the same striking effect, suggesting Hog1 independence. *RCK2* mutants share several phenotypes with negative regulators of PKA: cell lacking *RCK2*, *PDE2*, and the negative RAS/PKA regulator *IRA2* are sensitive to osmotic shock, while *rck2*Δ and *ira2*Δ mutants are resistant to the phosphodiesterase inhibitor caffeine (albeit detected in different studies [99, 100]). Intriguingly, Rck2 has sequence and functional homology with mammalian MAPKAP2, a stress activated kinase activated by the Hog1 ortholog p38 [101]. MAPKAP2 was recently shown to directly regulate Pde4 in human cells [102], supporting our conjecture that Rck2 regulates Pde2 and/or PKA in yeast as well.

A major challenge going forward in phospho-proteomic research is dissecting functional consequence of phosphorylation events. While some phosphorylation events produce major consequences in the cell, many likely fine tune dynamics, noise, and memory [103] and others may be inconsequential [17, 104]. Functional dissection of the outcome of such events (*e*.*g*. using phospho-mimicking mutations at specific sites) will be an important step in understanding cellular coordination during stress.

## Methods

### Strains and growth conditions

Strains used were of the BY4741 background unless otherwise noted [105] (Thermo Scientific, Waltham, MA). The *cdc14-3-URA3MX* mutant was kindly provided by Michael Tyers [106]. GFP-tagged strains were obtained from the yeast GFP collection [107] (Thermo Scientific, Waltham, MA). Correct knockouts or GFP integrations were verified by diagnostic PCR. Wild-type samples for phospho-proteomics were grown for at least 7 generations in YPD at 30°C to log phase, followed by collections before and 5 min (in biological triplicate), 15 and 30 min (in one replicate time course) after treatment with 0.7M NaCl. The *hog1*Δ and *pde2*Δ mutants, along with a paired wild type, were collected before and 5 min after NaCl treatment in biological duplicate. The *cdc14-3-URA3MX* strain and its parent, MT1901, were grown for 7 generations at 25°C followed by centrifugation for 2 min at 3K, decanting, and resuspension of cells in pre-warmed media at the non-permissible temperature (37°C). After 2 hr at the non-permissible temperature, cells were harvested before and 5 min after 0.7M NaCl treatment. Cdc14 inactivation was verified by a high fraction of dumbbell shaped cells [62]. Cells were collected by brief centrifugation, flash frozen, and stored at -80C.

### Co-immunoprecipitation (co-IP) assays

Hog1-GFP, Rck2-GFP, Cdc14-GFP, and Pde2-GFP were grown for at least 7 generations in YPD and harvested before and 10 min after 0.7M NaCl treatment, followed by immediate flash-freezing. BY4741 was included as an untagged control. Yeast cells were resuspended in lysis buffer (50 mM HEPES-KOH pH 7.5, 140 mM NaCl, 1 mM EDTA, 0.5% NP40, and 0.1% Na-Deoxycholate with protease inhibitors (Millipore, Billerica, MA) and phosphatase inhibitor NaF (Thermo Scientific, Waltham, MA)), and lysed by glass bead milling (Retsch, Newton, PA). Immunoprecipitations were performed by incubating ~12.5 mg of protein lysate with 25 μL of GFP-Trap MA beads (Chromotek, Germany) for 1.5 hr at 4°C. Beads were washed twice with 1 mL wash buffer (50 mM HEPES-KOH pH 7.5, 140 mM NaCl, 1 mM EDTA, 0.5% NP40), followed by 2 washes with 1 mL Tris wash buffer (150 mM NaCl, 10 mM Tris-CL pH 7.5, and 0.5 mM EDTA). Proteins were eluted with 20 **μl** 0.5% formic acid and lyophilized in a speed vacuum. Each mutant was interrogated in biological duplicate with a matched un-tagged wild-type strain as a control.

### Mass spectrometry summary

A brief overview of mass spectrometric analysis is provided here, with additional details in the supplement. Cell pellets were thawed on ice, washed twice with 1 ml ice-cold water, and resuspended in lysis buffer (8 M urea, 50 mM Tris pH 8.0, and protease and phosphatase inhibitor cocktail table, Roche, Indianapolis, IN) and rigorously vortexed. Yeast cells were lysed by glass bead milling (Retsch, Newton, PA). Briefly, 500 μl of acid washed glass beads were combined with 500 μl of resuspended yeast cells in a 2 ml Eppendorf tube and shaken at 4°C 8 times at 30 hz for 4 min with a 1 min rest in between. Bradford Protein Assay (Bio-Rad, Hercules, CA) was used to measure final protein concentration. Proteins were reduced by incubation for 45 min at 55°C with 5 mM dithiothreitol. The mixture was cooled to room temperature and alkylated by addition of 15mM iodoacetamide in the dark for 45 min. The alkylation reaction was quenched with equivalent amount of 5 mM dithiothreitol.

Cell lysates were prepared and diluted with 50 mM Tris to a final urea concentration of ~ 1.5 M before the addition of trypsin in 1:50 ratio (enzyme:protein; Promega, Madison, WI). Mixtures were incubated overnight at an ambient temperature, acidified by the addition of 10% TFA, desalted over a Sep-Pak (Waters, Milford, MA), and lyophilized to dryness in a SpeedVac (Thermo Fisher, Waltham, MA). Peptides were labeled with tandem mass tags (Pierce TMT, Rockford, IL), according to the manufacturer’s instruction. Labelled peptides were then mixed in 1:1 ratio, and the resulting mixture was desalted over a Sep-Pak. ~2.5 mg of the labelled peptide mixture were used to enrich for phospho-peptides via immobilized metal affinity chromatography (IMAC) using magnetic beads (Qiagen, Valencia, CA), according to the published method [108]. High pH reverse phase liquid chromatography was used to fractionate enriched phospho-peptides. Peptides were analyzed on a quadrupole-ion trap-Orbitrap hybrid Fusion or Fusion Lumos mass spectrometer (Thermo Scientific, San Jose, CA), as described in detail in S1 Supporting Information.

### Mass-spec data analysis

The raw data corresponding to TMT-labelled peptides were searched against *Saccharomyces* genome database (SGD) of yeast protein isoforms (downloaded 12.02.2014) and processed using the COMPASS software suite [109], with FDR correction at the peptide and protein level (<1%). TMT reporter region quantification was performed using an in-house software TagQuant, as previously described [110]. Briefly, the raw reporter ion intensity in each TMT channel was corrected for isotope impurities, as specified by the manufacturer for the used product lot, and normalized for mixing differences by equalizing the total signal in each channel. In cases where no signal was detected in a channel, the missing value was assigned with the noise level of the original spectrum (i.e. noise-band capping of missing channels), and the resultant intensity was not corrected for impurities or normalized for uneven mixing. The raw data corresponding to the Co-IP analyses were processed using MaxQuant (Version 1.5.2.8; [111]). Searches were performed against a target-decoy database of reviewed yeast proteins plus isoforms (Uniprot, downloaded January 20, 2013) using the Andromeda search algorithm with precursor search tolerance of 4.5 ppm and a product mass tolerance of 20 ppm, as further described in S1 Supporting Information (peptide and protein FDR < 1%). Proteins were identified by at least one peptide (razor + unique) and quantified using MaxLFQ with an LFQ minimum ratio count of 2. The ‘match between runs’ feature utilized, and MS/MS spectra were not required for LFQ comparisons.

To implicate co-IP’d interactors from contaminating proteins, we selected proteins that were i) identified in both biological IP replicates (either before or 10 min after NaCl treatment), and ii) were ≥2-fold more abundant in the GFP-tagged pulldown compared to the untagged control. Protein-protein interactions were subsequently classified as salt-dependent or salt-independent by calculating the log_2_ ratio of protein abundance at 10 min versus pre-stress (for this calculation, missing data was imputed as the lowest 1% of intensity scores from that run). Proteins with ≥1.5-fold differential abundance before versus after NaCl in both replicates were classified as salt-dependent interactions.

### Functional enrichments and true positive list

Unless otherwise noted, functional enrichments were calculated by the hypergeometric test using FunSpec [45], taking categories with P<1x10^-4^ (representing the Bonferroni-corrected threshold) as significant. A true positive list of genes involved in osmotic stress and signaling was constructed using the following approach. We started with a list of 110 ‘Osmotic stress response genes’ that were previously curated [25] as either HOG pathway components [55, 112], containing ‘osmotic’ or ‘osmolarity’ in their *Saccharomyces* Genome Database (SGD) annotation [54], or are annotated as ‘stress regulator’ and identified in at least one publication as osmotic stress associated. We also ran the literature mining tool Gadget (http://gadget.biostat.wisc.edu/) to identify yeast gene names associated with the terms “Osmotic Stress”, “Sodium Chloride” and “Stress” queries at FDR <0.05; we retained only genes encoding known regulatory proteins. Finally, we removed from the list genes encoding transcription factors, since these are not expected in the network that regulates the yeast phosphoproteome. This identified 70 proteins used as true-positive regulators of the yeast osmotic stress response.

### Computational pipeline

#### Defining phospho-peptide submodules

To identify changing phospho-peptides with significant phosphorylation change in replicate time points, we input count-based phospho-peptide intensities to edgeR [113] and took (FDR <0.05) as significant. In addition, we selected proteins with ≥1.5-fold change in 2 of the 3 paired replicates comparing to samples from paired, untreated cells. We added to this phospho-peptides from the single time course that had at least a 1.5X change in both the 15 and 30 min time points, or a single instance of ≥2X at the later time points. This identified 1,249 phospho-peptides that respond to NaCl, which were split into peptides that increased or decreased phosphorylation (S1 Table). Further partitioning based on hierarchical clustering of temporal profiles did not provide any benefits in identifying other clusters.

To identify reoccurring motifs, *motif-X* [47] was run separately on phospho-peptides with NaCl-dependent increases or decreases, using the following parameters: extend from: SGD yeast proteome; central character: s* or t*; width: 13; occurrences: 10; significance: 1x10^-6^. This yielded 17 motifs that capture 80% of all changing phospho-peptides. Groups were further split based on mutant dependencies as follows: for *hog1*Δ and *pde2*Δ mutants, peptides with ≥1.3-fold difference in abundance in both replicates (or 1.5-fold for peptides detected in only one of the two experiments) compared to wild type were considered affected in the mutant. Due to the well-known phenomenon of inference-induced ratio compression associated with TMT quantification, all measured changes were likely underestimated [114]; we therefore determined the aforementioned cutoffs manually so as to capture known targets of the regulators. Functional enrichments of the selected proteins support the approach: peptides identified as Hog1 dependent were enriched for ER to Golgi transport and cell growth, whereas Pde2 was enriched for cell growth, actin cytoskeleton, budding, cell polarity and filament formation, and guanine nucleotide exchange factors (GEFs) (S2 Table) (P < 1x10^-4^) [45]. Twenty-three percent of the proteins with Hog1-dependencies and found in the enriched groups require Hog1 for NaCl-responsive phosphorylation changes [21], validating our selection criteria. Fold-changes smaller in the mutant than the wild type were termed **defective**, changes that were greater in the mutant than wild type were termed **amplified**, and phospho-peptides that failed to meet these criteria were classified as having **no-phenotype** in the mutant. We relaxed the fold-change cutoffs for the *cdc14-3-URA3MX* mutant. This mutant exhibited smaller but still reproducible defects in phosphorylation compared to the other mutants, including for known Cdc14 targets and proteins linked to the cell cycle. The subtle but reproducible effects may be because Cdc14 functions in only a subset of cells in the population, other phosphatases act partially redundantly, or the required experimental procedure limited our power to detect defects. We therefore used relaxed criteria to identify phospho-peptides affected by Cdc14, requiring a reproducible 1.15-fold defect in phosphorylation compared to the mock-treated wild type, or a 1.3-fold defect for peptides detected in a single sample. Phospho-peptides identified as Cdc14-affected were enriched for functions related to Cdc14 activity, including cell cycle, budding, cell polarity and filament formation, in addition to GTPase signal transduction and kinase activity (S2 Table). Together, these criteria were used to subdivide each of the 17 modules into 76 submodules. Peptides affected by multiple mutants were represented in each of the corresponding submodules, rather than placed into submodules with multi-mutant effects, due to small numbers.

#### Identification of shared interactors and phospho-motif matches

We used a background physical protein-protein interaction (PPI) network as compiled by Chasman *et al* [25], which included protein-protein and kinase-substrate interactions measured in multiple high-throughput studies (or individual low-throughput studies), compiled from various databases [52, 53, 106, 115, 116]. To ensure that only high-confidence interactions were included, we required that interactions from high-throughput datasets were identified by at least two, separate experimental methods. PPIs were predominantly sourced from the BioGRID database [53] and included non-directed, PPI interactions and directed, post-translational modifications, including phosphorylation. To maximize inclusion of signaling pathways, the background network was supplemented with high-confidence, directed, kinase-substrate and phosphatase-substrate interactions obtained from the KID database [52] and data from Ptacek *et al* [65]. In total, the background network contained 4,638 nodes, representing proteins, and 25,862 interactions, of which 19,921 were non-directed and 5,761 were directed.

For each submodule of peptides, we identified proteins from the PPI network that showed more interactions with submodule constituent proteins than expected by chance (hypergeometric test, FDR < 0.05), which yielded 472 SIs linked to one or more of 54 submodules. Edge directionality was determined based on known kinase-substrate relationships: **input** edges from the SI to the submodule were assigned for SIs with at least one directed interaction with submodule constituents or for SIs with known, non-directional interactions with those constituents. We classified SIs with non-directional interactions as inputs because these interactions might be directed, but were not identified as such because of previous experimental design limitations or tested conditions. In contrast, directed edges from the submodule to the SI were defined as **output** edges if all of interactions were directed toward the SI; these edge directionalities were considered in the subsequent ILP.

For final network visualization, we categorized edges as **motif-match** if the known specificity of its SI kinase matched the submodule motif identified by *motif-X*. We first generated a position weight matrix (PWM) for module peptides distinguished by *motif-X*, adding a pseudocount for each amino acid to prevent overfitting (S9 Table). These were compared to protein-array defined specificities for 63 kinases from Mok *et al* [49], containing background-corrected phosphorylation signal intensities that were normalized to total signal intensities for all amino acids. We converted to frequencies by summing signal intensities for all amino acids at a position (after addition of a pseudocount) and dividing by the summed intensities. Adding a pseudocount was necessary as some amino acids were not detected in the protein-array, and Kullback-Leibler Divergence (KLD) requires non-null values at all positions. Next, we compared the module PWMs to the adjusted Mok PWMs using Kullback-Leibler Divergence. To assess statistical significance of the matches, we generated a randomized distribution of KLD scores by permuting within-column values, and then shuffling the columns for each kinase PWM from [49] 1000 times, generating 63,000 KLD scores per module. FDR was taken as the number of randomized KLD scores that had a smaller KLD distance than the observed value.

A motif-match designation was assigned to kinases with the smallest FDR scores that also belong to a kinase group (as assigned by Mok *et al*) that is known to recognize the module motif. Kinases that were not in the Mok *et al* dataset were classified as having **unknown-recognition motif** relationships to all modules.

#### Integer linear program method for subnetwork inference

We use an ILP to select paths through the modified background network to link interrogated source regulators to their downstream phospho-peptide submodules, minimizing the number of intermediate nodes used by all paths while maximizing the inclusion of shared interactors (SIs). The ILP is a modified version of what was proposed by Chasman *et al* [25]. An overview of the approach is described below with a detailed description provided in S1 Supporting Information Section 2.

We first augmented the background network with 1,835 edges capturing SI-to-submodule *input* edges and submodule-to-constituent or submodule-to-SI *output* edges as described above (S5 Table). We next enumerated all possible acyclic paths of up to 3 edges (discounting submodule-to-constituent edges), between interrogated *source* regulators (Hog1, Pde2, Cdc14) and the phospho-peptide submodules that exhibit a phenotype, executed as a depth-first search through the background network. Submodules without any mutant dependencies were included as nodes in the background network and may appear as intermediates in paths but not as termini. We assign a binary variable to each network element (node, edge, and path) to represent whether the element is selected for inclusion in the subnetwork or not. For undirected edges, we also assign a directionality variable, *d* which is set to 1 if the edge is selected in the ‘forward’ direction (determined by lexicographic order of the node names) and 0 otherwise. We also make use of a variable *c_s,m_* for each source-submodule pair that indicates whether the pair has been connected by a selected path.

We developed a multi-part objective procedure similar to our previous work [25] and described in detail in S1 Supporting Information Section 2. At each step, the result of the solution is added to the ILP as an additional constraint that must be satisfied during the next optimization step: 1. Maximize the number of source-submodule pairs connected by a selected path. 2. Find the maximum number of SI edges that can be included in a valid subnetwork. 3. Minimize the number of intermediate nodes that are not *sources*, *submodules*, or SIs. 4. Maximize the number of selected paths. We implemented a step to increase diversity in the final solutions based on a previous approach [50]. Specifically, after each solution we randomly hold aside (that is, fix its *y* variable to 0) with 5% probability all non-source, non-submodule nodes from the previous solution. As a result, about 5% of previously selected nodes are held aside in each iteration. We repeated this procedure 1000 times. After pooling these solutions, we defined a consensus network based on source-submodule paths found in at least 75% of all solutions. As a final step, we added to this consensus all submodules without mutant phenotypes whose SI was included in the consensus, with a direct edge emerging from its SI to that submodule. In all, the final network included 218 proteins in regulator paths and 51 submodules (together capturing 832 of 1249 phospho-peptides), with 844 edges between them. Precision-recall analysis is described in S1 Supporting Information Section 3.

## Supporting information

S1 Supporting Information The supporting information document includes supplemental methods and additional validation studies of the inferred subnetwork. (DOCX)

S1 Fig Precision recall curves comparing ILP from real versus scrambled PPI data. As shown in Fig 2B, the ILP from real data had significantly higher precision and recall of a list of true positive regulators activated by NaCl. The ILP networks from scrambled PPI data also showed significantly lower enrichment for kinases (*P* from 3.5x10^-12^ to 3x10^-08^) compared to the real ILP network (*P* = 3.5x10^-36^), and lower enrichment for proteins that interact with Hog1 or are in the HOG pathway (*P* from 9.1x10^-07^ to 0.07) compared to the real ILP network (*P* = 8.4x10^-26^). We also compared the collection of SIs identified from real PPI data versus SIs identified from 1,000 scrambled PPI networks. Analyzing the collection of SIs identified in each case, none of the 1,000 randomized trials reached the same precision or recall as real data. We also scored the fraction of times that Hog1 was connected to Hog1-dependent submodules. In the network generated from real PPI data, Hog1 was an SI to three submodules; two of these (67%) matched submodules whose phospho-motifs matched the known Hog1 specificity. In 88% of the randomized trials, Hog1 was never matched to a submodule with the known Hog1 specificity. In 3% of randomized trials, Hog1 was matched to 3 submodules but only 1 matched Hog1 specificity. Thus, while some of the ILP network features are influenced by structure in the PPI background network, many of the insights uncovered here are due to real biology and not simply underlying network structure.
(TIF)

S2 Fig Examples of loop structure in PathLinker networks. A) Examples of looping seen in the PathLinker *k* = 200 network inferred for the Pde2 source. These examples were manually chosen for nodes connected to Tpk1 or Hog1. B-C) Degree distribution for the B) ILP (75% confidence) network and the C) Pathlinker *k* = 200 Pde2 network. PKA subunits (pink nodes), Hog1 (purple node), and proteins involved in transcriptional regulation (orange node).
(TIF)

S1 Table Fold-changes for phospho-peptides reproducibly changing in response to NaCl treatment. (XLSX)

S2 Table Fold-changes for phospho-peptides with mutant dependencies during NaCl stress. (XLSX)

S3 Table Gene ontology enrichment for phospho-peptides with mutant dependencies. (XLSX)

S4 Table Submodule constituent proteins and phospho-sites. (XLSX)

S5 Table Experimentally determined edges added to the background network. (XLSX)

S6 Table Gene ontology enrichments for proteins captured in the transcriptional, phosphorylation, or overlap of the networks. (XLSX)

S7 Table Co-immunoprecipitation results. (XLSX)

S8 Table Gene ontology enrichments for proteins captured in repressed submodules connected to PKA. (XLSX)

S9 Table Position weight matrices representing amino acid prevalence for each module. (XLSX)

S1 Dataset Cytoscape session containing the consensus phosphorylation network at 75% confidence. (ZIP)
