# Supplementary material for: Network inference reveals novel connections in pathways regulating growth and defense in the yeast salt response
Source: PLoS Comput Biol. 2018 May 8;13(5):e1006088. doi: 10.1371/journal.pcbi.1006088 (PMC5940180; doi:10.1371/journal.pcbi.1006088)
Supplement: S1 Supporting Information — (DOCX) [file pcbi.1006088.s001.docx]

S1 SUPPORTING INFORMATION

Contents

1. **Phospho-proteomic methods**
   1. **Tryptic digest, TMT labelling, phospho-peptide enrichment, and fractionation**
   2. **Preparation of Co-IPs for LC/MS-MS analysis**
   3. **LC-MS/MS**
   4. **Mass spec data analysis**
2. **Detailed computational methods**
   1. **Integer linear program (ILP) method for subnetwork inference**
   2. **Background network**
   3. **Constraints define a valid directed subnetwork**
   4. **Optimization for inclusion of shared interactors**
   5. **Obtaining an ensemble of 1000 subnetworks**
   6. **Define a high-confidence consensus subnetwork**
   7. **Adding back no-phenotype submodules to the consensus network**
   8. **Comparison to solution pool-based ensemble strategy from previous work**
   9. **Network Randomization**
   10. **Prize-collecting Steiner forest comparison**
   11. **PathLinker comparison**
   12. **Precision-recall and receiver operating analysis**
3. **Computational validation supports the inferred subnetwork**

**1. Phospho-proteomic methods**

**a. Tryptic digest, TMT labelling, phospho-peptide enrichment, and fractionation**

Cell pellets were thawed on ice, washed twice with 1 ml ice-cold water, and resuspended in lysis buffer (8 M urea, 50 mM Tris pH 8.0, and protease and phosphatase inhibitor cocktail table, Roche, Indianapolis, IN) and rigorously vortexed. Yeast cells were lysed by glass bead milling (Retsch, Newton, PA). Briefly, 500 µl of acid washed glass beads were combined with 500 µl of resuspended yeast cells in a 2 ml Eppendorf tube and shaken at 4ºC 8 times at 30 hz for 4 min with a 1 min rest in between. Bradford Protein Assay (Bio-Rad, Hercules, CA) was used to measure final protein concentration. Proteins were reduced by incubation for 45 min at 55 °C with 5 mM dithiothreitol. The mixture was cooled to room temperature and alkylated by addition of 15mM iodoacetamide in the dark for 45 min. The alkylation reaction was quenched with equivalent amount of 5 mM dithiothreitol. Lysates were diluted with 50 mM Tris to a final urea concentration of ~ 1.5 M before the addition of trypsin in 1:50 ratio (enzyme:protein; Promega, Madison, WI). Mixtures were incubated overnight at an ambient temperature, acidified by the addition of 10% TFA, desalted over a Sep-Pak (Waters, Milford, MA), and lyophilized to dryness in a SpeedVac (Thermo Fisher, Waltham, MA). Peptides were labelled with tandem mass tags (Pierce TMT, Rockford, IL), according to the manufacturer’s instruction. Labelled peptides were then mixed in 1:1 ratio, and the resulting mixture was desalted over a Sep-Pak. ~2.5 mg of the labelled peptide mixture were used to enrich for phospho-peptides via immobilized metal affinity chromatography (IMAC) using magnetic beads (Qiagen, Valencia, CA), according to the published method [1]. Flow through unmodified peptides were lyophilized to dryness, resuspended in 0.2% formic acid, and ~2 µg of peptides were analyzed unfractionated.

High pH reverse phase liquid chromatography was used to fractionate enriched phospho-peptides. Mobile phase A consisted of 20 mM ammonium formate pH 10.0; mobile phase B contained 80% acetonitrile in 20 mM ammonium formate pH 10.0. Phospho-peptides were fractionated into 16 factions, concatenated into 8 combined fractions, lyophilized to dryness, and each fraction was resuspended in 15 µl 0.2% formic acid for LC-MS/MS analysis. Two injection replicas of 5 µl were analyzed via LC-MS/MS analyses.

**b. Preparation of Co-IPs for LC-MS/MS analysis**

Co-IPs were resuspended in 50 µl lysis buffer (8 M urea, 100 mM Tris pH 8.0, 10 mM TCEP, and 40 mM 2-chloracetamide) and vortexed for 10 min. Proteins were then precipitated by the addition of methanol to the final concentration of 90% and pelleted by centrifugation at 14,000 g for 45 min. After decanting supernatant resulting protein pellets were resuspended in the same lysis buffer and digested as described above, with the exception that 10 µg StrataX solid phase extraction columns (Phenomenex, Torrance, CA) were used for desalting. Peptides were resuspended in 25 µl 0.2% formic acid, and 5 µl of each sample were used in the LC-MS/MS analyses.

**c. LC-MS/MS**

All capillary columns were prepared in house, as previously described [2]. Briefly, a laser puller (Sutter Instruments Co., Novato, CA) was used to generate 75–360 µm inner-outer diameter bare-fused silica capillary columns with electrospray emitter tips. The tip was plugged with ~5 mm of 5 µm, 130 Å pore size, Bridged Ethylene Hybrid (BEH) C_18_ particles (Waters, Milford, MA). Columns were packed with 1.7 µm diameter BEH particles to a final length of ~30 cm and installed on a Dionex Ultimate 3000 nano HPLC system (Thermo Fisher, Sunnyvale, CA), using a stainless steel ultra-high pressure union (IDEX, Oak Harbor, WA). All used solvents were LC-MS grade and purchased from Thermo Fisher Scientific (Waltham, MA). Mobile phase buffer A consisted of 0.2% formic acid water; mobile phase B consisted of 70% acetonitrile, 0.2% formic acid in water, and 5% DMSO. Columns were heated to 60-65°C inside an in-house made heater. Peptides were loaded onto a column in 0% B and separated at a flow rate of 300-400 nl/min over a 90 min gradient for phospho-peptides and 60 min gradient for co-IPs.

Eluting peptides were analyzed on a quadrupole-ion trap-Orbitrap hybrid Fusion or Fusion Lumos mass spectrometer (Thermo Scientific, San Jose, CA). Orbitrap survey scans were performed at a resolving power of 60,000 at 200 m/z with an AGC target of 1x10^6^ ions and maximum injection time set to 100 ms. The instrument was operated in the Top Speed mode with 2 s cycles and monoisotopic precursor selection turned on. All tandem MS/MS scans were acquired on precursors with charge states of 2-6 with ion count target was set to 5x10^4^. For TMT-labelled samples tandem scans were collected in the Orbitrap at a resolving power of 60,000 at 200 m/z using HCD fragmentation with normalized collision energy of 35, dynamic exclusion of 25 s, and the maximum injection time of 350 ms. For co-IPs tandem MS scans were collected in the Orbitrap at a resolving power of 15,000 at 200 m/z using HCD fragmentation with normalized collision energy of 25, dynamic exclusion of 20 s, and maximum injection time of 25 ms.

**d. Mass spec data analysis**

The raw data corresponding to TMT-labelled peptides were searched against *Saccharomyces* genome database (SGD) of yeast protein isoforms (downloaded 12.02.2014) and processed using the COMPASS software suite [3]. Carbamidomethylation (+57.0513 Da) of cysteine residues and TMT 10plex (+229.1629 Da) on N-termini of proteins and lysine residues were included as fixed modifications. Oxidation of methionine (+15.999 Da) and TMT 10plex on tyrosine (+229.1629 Da) were included as variable modifications. Average mass tolerances of 125 ppm and 0.015 Da were allowed for MS1 precursor searches and MS2 fragment searches, respectively. Up to 3 missed cleavages on tryptic peptides following the proline rule were allowed. 1% false discovery rate (FDR) correction was performed on all identified peptides and proteins. TMT reporter region quantification was performed using an in-house software TagQuant, as previously described [4]. Briefly, the raw reporter ion intensity in each TMT channel was corrected for isotope impurities, as specified by the manufacturer for the used product lot, and normalized for mixing differences by equalizing the total signal in each channel. In cases where no signal was detected in a channel, the missing value was assigned with the noise level of the original spectrum (i.e. noise-band capping of missing channels), and the resultant intensity was not corrected for impurities or normalized for uneven mixing.

The raw data corresponding to the Co-IP analyses were processed using MaxQuant (Version 1.5.2.8; [5]). Searches were performed against a target-decoy database of reviewed yeast proteins plus isoforms (Uniprot, downloaded January 20, 2013) using the Andromeda search algorithm with precursor search tolerance of 4.5 ppm and a product mass tolerance of 20 ppm. Search parameters included fixed modification for carbamidomethylation of cysteine residues and a variable modification for the oxidation of methionine and protein N-terminal acetylation, and a maximum of 2 missed tryptic cleavages. A 1% peptide false discovery rate (FDR) and a 1% protein FDR were applied. Proteins were identified by at least one peptide (razor + unique) and quantified using MaxLFQ with an LFQ minimum ratio count of 2. The match between runs feature was turned on, and MS/MS spectra were not required for LFQ comparisons.

**2. Detailed computational methods**

**a. Integer linear program (ILP) method for subnetwork inference**

We use an integer linear program (ILP) to selects paths through the modified background network to link interrogated source regulators to their downstream phospho-peptide submodules, minimizing the number of intermediate nodes used by all paths while maximizing the inclusion of shared interactors (SIs). The ILP is a modified version of what was proposed by Chasman *et al* [6].

**b. Background network**

We augmented the background network with 1,835 edges capturing SI-to-submodule input edges and submodule-to-constituent protein edges. Each edge was assigned one of the following labels: **Constituent** interactions are represented by an outgoing directed edge from each submodule to each constituent protein. Kinase SIs with edges directed toward submodules (**inputs**) were classified either as **motif-match**, **motif-unmatch**, or **unknown recognition motif** edges based on the KLD analysis described in Methods. Directionality for **Shared interactor** edges was defined as described in Methods, either as **input** to the submodule or **output** from the submodule if there was *a priori* information supporting the directionality. Of these 1,835 edges, 899 were constituent edges, 679 shared interaction edges, 77 kinase motif-match edges, 98 kinase motif-unmatched edges, 67 kinase unknown recognition motif edges, 11 phosphatase motif-unmatched edges, and 4 were output edges (Table S5).

**Step 1. Define candidate paths**

We start by enumerating all possible acyclic paths of up to 3 edges (discounting submodule-to-constituent edges), between interrogated **source** regulators (Hog1, Pde2, Cdc14) and the phospho-peptide submodules that exhibit a phenotype. This enumeration is executed as a depth-first search through the background network. Submodules without any mutant dependencies were included as nodes in the background network and may appear as intermediates in paths, but not as termini.

**Step 2. Infer an ensemble of subnetworks linking sources and shared interactors to submodules**

**Notation**

The input network to the approach is represented as a graph of nodes $\mathcal{N}$ (including proteins and submodules), edges $\mathcal{E}$, and candidate paths $\mathcal{P}$, where each path is an ordered set of nodes and edges. We also define subsets of nodes, edges, and paths. We define the subset of source nodes as $\mathcal{N}^{S}$ and the submodules as $\mathcal{N}^{m}$. Edges include undirected edges $\mathcal{E}^{U}$, directed edges $\mathcal{E}^{D}$, and edges between shared interactors and submodules $\mathcal{E}^{SI}$.

**Variables**

We assign a binary variable to each network element (node, edge, and path) to represent whether the element is selected for inclusion in the subnetwork or not. These selection variables are $y$ (nodes), $x$ (edges), and $\sigma$ (paths). For undirected edges, we also assign a directionality variable,$d$ which is set to 1 if the edge is selected in the ‘forward’ direction (determined by lexicographic order of the node names) and 0 otherwise. We also make use of a variable $c_{s,m}$ for each source-submodule pair that indicates whether the pair has been connected by a selected path.

**c. Constraints define a valid directed subnetwork**

The following constraints define a subnetwork, which is a set of directed paths that connect source-submodule pairs. Most constraints come in pairs, where one constraint provides an upper bound on a selection variable, and the other provides a lower bound.

**For a source-submodule pair to be connected, there must be at least one selected path that includes both source and submodule**

The set of paths from source $s$ to one of its submodules $m$ is referred to as $\mathcal{P}\left( s,m \right).$ Because we will be maximizing the number of connected pairs, there is no need to define a constraint to provide a lower bound.

| $c_{s,m}\leq\sum_{p \in\mathcal{P}\left( s,m \right)} \sigma_{p}$ | For each source-submodule pair *(s,m)* | (1) |
| --- | --- | --- |

**To select a path, we must also select all of its edges**

We refer to the edges in path $p$ as $\mathcal{E}\left( p \right)$.

| $\sigma_{p}\leq x_{e}$ | For all $p\in\mathcal{P}$, $e\mathcal{\in E}\left( p \right)$ | (2) |
| --- | --- | --- |

**To select an edge, it must be in a selected path**

| $x_{e}\leq\sum_{p\in\mathcal{P}\left( e \right)} \sigma_{p}$ | For all $e\mathcal{\in E}$ | (3) |
| --- | --- | --- |

**To select an edge, we must also select its constituent nodes.**

We refer to the set of nodes involved in the edge $e$ as $\mathcal{N}\left( e \right)$

| $x_{e}\leq y_{n}$ | For all $e\mathcal{\in E,}n\in\mathcal{N}\left( e \right)$ | (4) |
| --- | --- | --- |

**A selected node must be part of at least one selected edge**

For a node $n$, we refer to its set of edges as $\mathcal{E}\left( n \right)$

| $y_{n}\leq\sum_{e\mathcal{\in E}\left( n \right)} x_{e}$ | For all $n\in\mathcal{N}$ | (5) |
| --- | --- | --- |

**A path can only be selected if its undirected edges are oriented from *source* to submodule**

We uniquely assign a direction to each selected undirected (PPI) edge such that paths that it participates in proceed from *source* to *submodule*. During enumeration of paths, we record which direction of an edge will orient the path properly and refer to it as$dir(p,e)$. The indicator function$I(d_{e}=dir\left( p,e \right))$ returns true if the edges are oriented to satisfy the direction of the path.

| $\sigma_{p}\leq I(d_{e}=dir\left( p,e \right))$ | For all $p\in\mathcal{P}$, $e \in\mathcal{E}^{U}\left( p \right)$ | (6) |
| --- | --- | --- |

**d. Optimization for inclusion of shared interactors**

In order to identify an optimal subnetwork that reveals connections between sources, submodules, and shared interactors using a minimum number of intermediate nodes not supported by evidence, we developed a multi-part objective procedure similar to what was used in the previous work [6]. At each step, the result of the solution is added to the ILP as an additional constraint that must be satisfied during the next optimization step.

1. **Maximize the number of source-module pairs that are connected by a selected path.** The purpose of this step is to define a subnetwork that connects as many as possible sources to their affected submodules. We call the maximum number of source-module connections $M^{C}.$

| $M^{C}=max \sum_{\boldsymbol{source-submodule pairs (s,m)}} c_{\boldsymbol{s,}\boldsymbol{m}}$ | (7) |
| --- | --- |

After optimizing this quantity, we add a new constraint to the ILP:

| $\sum_{\boldsymbol{source-submodule pairs (s,m)}} c_{\boldsymbol{s,m}}\boldsymbol{=}M^{C}$ | (8) |
| --- | --- |

1. **Find the maximum number of SI edges that can be included in a valid subnetwork.** This step optimizes the inclusion of edges between proteins and modules for which they are enriched for physical interactions. We call the maximum number of included SI edges $M^{SI}.$

| $M^{\mathrm{SI}}=max \sum_{\boldsymbol{e\in}\mathcal{E}^{SI}.} x_{\boldsymbol{e}}$ | (9) |
| --- | --- |

After optimizing this quantity, we add a new constraint to the ILP:

| $\sum_{e\in\mathcal{E}^{SI}.} x_{e}=M^{\mathrm{SI}}$ | (10) |
| --- | --- |

1. **Minimize the number of intermediate nodes that are not sources, submodules, or involved in SIs.**

| $\min\sum_{n\in\mathcal{N-}\mathcal{N}^{S}-\mathcal{N}^{M}\mathcal{-N(}\mathcal{E}^{SI})} y_{n}$ | (11) |
| --- | --- |

After this step, the selection variables *y* for all nodes are fixed to their current levels.

1. **Maximize the number of selected paths.** The final objective function serves to reveal all possible directed paths among selected nodes. Without this step, paths and edges may be arbitrarily chosen by the solver.

| $\max\sum_{p\in\mathcal{P}} \sigma_{p}$ | (12) |
| --- | --- |

**e. Obtaining an ensemble of 1000 subnetworks**

There are multiple subnetworks that are optimal according to the objective functions. To quantify the importance of network features (nodes, edges, paths) for connecting the sources, SIs, and submodules, we obtained an ensemble of subnetworks. To do so, we developed a procedure where the entire optimization procedure is applied many times sequentially, and each solution is required to give a subnetwork for which the intermediate node set is at least somewhat different from the previous solution. After each solution, we take the set of non-source, non-submodule nodes that were selected and randomly choose to hold each aside (that is, fix its *y* variable to 0) with 5% probability. As a result, about 5% of previously selected nodes are held aside in each solution. We repeated this procedure 1000 times. Our approach was motivated by a previously described algorithm for finding diverse solutions to binary ILPs [7], which sequentially identifies solutions that are maximally different from solutions that have already been found.

**f. Define a high-confidence consensus subnetwork**

We used the ensemble of subnetworks to quantify confidence in node, edge, and path as the fraction of subnetworks in which they were selected. To define the consensus subnetwork, we took all source-submodule paths with at least 75% confidence.

**g. Adding back no-phenotype submodules to the consensus network**

Since the ILP only allows incorporation of no-phenotype submodules if they serve as intermediate nodes, a subset of no-phenotype submodules were not captured in the consensus network. We added back all no-phenotype submodules that had SIs represented in the consensus network, and directed the edge based on the SI-submodule pair input/output classification. Implementing this approach, all no-phenotype submodules were included in the consensus network through the addition of 141 SI-no-phenotype submodule edges. Supplementary Dataset S1 contains the consensus network (at 75%) as a Cytoscape [8] v3.2.1 session.

**h. Comparison to solution pool-based ensemble strategy from previous work**

In the previous manuscript [6], we obtained an ensemble of subnetworks using the CPLEX commercial solver Solution Pool functionality, which collects additional optimal solutions that are identified in the solution tree during the solution process. We compared our new strategy (the ‘lesion’ approach) to the ‘solution pool’ approach on the basis of the relevance of additional non-SI proteins that were captured by the consensus subnetworks; that is, proteins that were not preferred by the objective functions. Although the subnetworks were largely similar, the consensus subnetwork from the lesion approach captured known salt response proteins (as described in Result section ‘Phospho-proteome network inference’) at a modestly higher rate than the pool approach. Therefore, we used the lesion consensus subnetwork for further analysis because it might be useful for generating interesting additional hypotheses about known relevant proteins based on their predicted interactions.

**i. ILP networks generated from scrambled background networks**

We used the R package BiRewire [9] to scramble the input PPI network using the function “rewire.bipartite” to produce 1,000 networks. BiRewire performs the minimum number of switching steps that promote maximized dissimilarity between the randomized and original network, while preserving node degree [9]. The scrambled background networks were analyzed as described in S1 Fig, and five were used as input for our computational pipeline, which was run according to the steps outlined in Methods.

**j. Prize-collecting Steiner forest comparison**

We compared the ILP with the prize-collecting Steiner forest (PCSF) algorithm, an established subnetwork inference method that connects a set of prize nodes to a set of source nodes. We used the Omics Integrator (version 0.3.0) [10] implementation of PCSF and msgsteiner (version 1.3) [11] for optimization. Prize nodes receive positive scores and PPI edges receive positive costs. The PCSF objective is to identify a tree or a forest (a collection of disjoint tree-structured graphs) subnetwork that connects the prize nodes to the source nodes such that the total scores of the included prize nodes are maximized and the edge costs of the included PPI are minimized. By rewriting the preference to maximize included prizes as a preference to minimize excluded prizes, the objective can be expressed as

| $\underset{F}{\mathrm{argmin}} \sum_{v\notin V_{F}} \left( \beta*p(v)-\mu*degree(v) \right)+0.01*\left\vert E_{F} \right\vert$ | (1) |
| --- | --- |

This objective function is a simplified version of the general PCSF objective [9] because in this application the node prizes are constant, edge costs are constant, and only one source node is considered at a time. The forest $F$ is the identified subnetwork. The first term in the objective sums over vertices (proteins) $v$ included in the forest vertex set $V_{F}$. Each vertex $v$ has a derived prize that is a function of hyperparameters $\beta$ and $\mu$ as well as the original prize $p(v)$ and the vertex degree, the number of neighboring vertices in the PPI graph. Larger $\beta$ encourages larger PCSF subnetworks. Larger $\mu$ penalizes high-degree hub nodes to a greater degree, discouraging their inclusion in $V_{F}$. The second term in the objective is the cumulative cost of the set of edges $E_{F}$ included in the forest. In general, each edge could have a unique cost. In this application, costs are all 0.01 (the minimum allowed in the Omics Integrator software).

PCSF cannot enforce the requirement that specific prize nodes are connected to specific source nodes. Therefore, we ran PCSF independently once per source node (Cdc14, Hog1, Pde2) and merged the results. In each run, we assigned the submodule vertices that correspond to the source a prize $p(v)$ of 1.0 and all other submodules for the other phenotypes a prize of 0. SI nodes receive no special designation. For each source, we created an ensemble of subnetworks by sweeping over values for $\beta$ and $\mu$ and rerunning the method 10 times per parameter combination with a different random seed. We tested $\beta$ values of 0.1, 0.5, 1.0, 1.5, 2.0, 2.5, 3.0, 3.5, 4.0, 4.5, and 5.0. We tested $\mu$ values of 0, 0.01, 0.02, 0.03, 0.04, 0.05, 0.06, 0.07, 0.08, 0.09, and 0.1. The final ensemble contained 3630 subnetworks: 3 sources × 11 values of $\beta$ × 11 values of $\mu$ × 10 random seeds. However, 1877 subnetworks were discarded because they were empty, which happens when $\beta$ is too small or $\mu$ is too large relative to the other parameters and inputs.

We treated the node frequency in the ensemble as a proxy for confidence, similar to the ILP, but did not filter the subnetwork based on frequency. Constructing the ensemble from multiples values of $\beta$ and $\mu$ instead of selecting a single parameter combination can improve the PCSF node ranking. The most important nodes appear in the PCSF solutions for many parameter combinations and are assigned the greatest confidence. In addition, tree-structured graphs can have poor recall because they only include one path to each prize node, which can be alleviated by combining many diverse PCSF solutions.

**k. PathLinker comparison**

We also compared the ILP with PathLinker (version 1.1), an algorithm for identifying pathways by connecting sources and targets in a biological network [12]. PathLinker takes a list of source nodes and target nodes as input and computes the *k* weighted shortest simple paths from a source to a target. Simple paths are source-target paths that do not visit any node more than once. PathLinker operates on a weighted, directed network; however, PPI networks are not directed. We therefore transformed all undirected edges in our background network to a pair of directed edges as recommended. We used PathLinker’s PageRank [13] preprocessing step to compute edge weights. This preprocessing first executes a random walk with restarts to derive node visitation probabilities. Then, it normalizes each node’s visitation probability by its degree to obtain a weight for all of the node’s outgoing directed edges.

Like PCSF, PathLinker does not support matching targets with specific sources if several sources are given. Therefore, we ran PathLinker separately for each source node and merged the three outputs. PathLinker output a list of edges ranked by the index of the first (best) source-target path that contains the edge. To merge the three source-specific outputs for subnetwork visualization and evaluation, we computed the minimum edge rank for each edge. To rank nodes for the PathLinker precision-recall evaluation, we computed the node’s minimum edge rank over all edges that contain that node.

PathLinker uses *k*, the number of shortest paths, to control the size of the subnetwork. Because networks for small *k* are subsets of networks for larger *k*, we ran PathLinker with *k* = 100,000 and then evaluated various thresholds by truncating the ranked edge lists at smaller *k*. We ultimately selected *k* = 100 for our primary comparison with the ILP because it produced a subnetwork comparable in size to the ILP’s subnetwork.

**l. Precision-recall and receiver operating characteristic analysis**

We calculated precision-recall (PR) curves for the ILP, PCSF, and PathLinker consensus networks, and for five ILP networks generated from randomized background networks as input, using the R library PRROC, which takes as input two vectors [14]. The first vector was a confidence score for each protein in the background network that indicates how often it was found in the network ensemble solution. Confidence scores ranged from 0 to 1, with 0 representing no inclusion in the ensemble and 1 representing inclusion in all solutions. The second vector was either a 0 or 1, indicating the presence or absence of a protein from a true positive (TP) list, described in Methods, Functional enrichments and true positive list. The TP list consisted of 70 curated signaling proteins known to be involved in the osmotic or stress response in yeast. Submodules were excluded from these analyses. To benchmark the predictive accuracy of the ILP method we compared the area under the curve (AUC) between the PR curves generated for consensus networks from each method, according to the method of Davis & Goadrich [15]. Random classifier plots were generated by PRROC [14]. *Precision* is the fraction of retrieved proteins that are on the given true positive list, while *Recall* is the fraction of true positives on that list that are retrieved.

**3. Computational validation supports the ILP inferred subnetwork**

Our computational approach outperformed two other established procedures.

NetworKIN is a popular method that predicts kinases physically connected directly to, or one interaction removed from, peptides whose phosphorylation motif matches the known kinase preference. However, it has several limitations; it cannot capture networks, it requires a previously observed interaction between kinases and each target, and it only reports on kinases with known phosphorylation motifs. Our method outperformed NetworKIN on several levels. The kinase-SIs identified by our approach were enriched for osmosensing and stress response (*P* < 1x10^-04^), whereas the NetworKIN output was not [22]. Furthermore, only our method captured known HOG kinases Ste11, Ssk2, Ssk22, and Pbs2, critical for the osmotic stress response [23, 24]. Beyond this, our method identified significantly more SIs that are kinases (71 versus 54) and predicted regulators for 54 submodules compared to 50 for NetworKIN. Finally, as mentioned above, NetworKIN does not produce a network but rather predicts regulators of submodules, a major limitation in predicting regulatory control.

Our approach also outperformed the PCSF method described above [10]. The ILP was better at capturing proteins from a true positive list (Fig 2). The AUC was ~ 1.2-fold greater for the ILP vs. the PCSF-generated consensus networks. The ILP is designed to use an unweighted network and PathLinker implements a pre-processing step to derive edge weights for unweighted graphs. In contrast, PCSF works best with weighted graphs and node prizes that reflect target node importance. The unweighted nodes and edges in our study may be responsible for its lower AUC.

Furthermore, our ILP captured more submodules than PCSF (46 versus 37, before addition of no-mutant-phenotype submodules not included during network inference, see Methods). Unlike PCSF, the ILP explicitly rewards including SI nodes in its subnetwork, which enables it to capture a greater number of putative regulators of the differentially phosphorylated phospho-peptides (Fig 2, Table 1). The ILP recovered 30% of all SIs (143/472) in comparison to 19% (91/472) in the PCSF subnetwork (Table 1). Of these SIs, the ILP identified 71 unique SI kinases and 5 unique SI phosphatases, compared to 21 kinases and 2 phosphatases for PCSF. Taken together, these results highlight the predictive accuracy of the consensus network and the advantages of our custom, multi-criteria objective function.

For subnetwork sizes greater than or equal to 100, PathLinker and the ILP have nearly identical precision and recall. The main difference between the two precision-recall curves is that PathLinker assigns almost all nodes a distinct rank, enabling it to prioritize its top 100 nodes better than the ILP, which assigns the top 100 nodes the maximal confidence. This early precision leads to PathLinker’s higher AUC, as does the longer list of ranked nodes when it is run with larger *k*.

The ILP outperformed PathLinker by exhibiting better predictive power for SIs and submodules. Although both methods are designed to link targets to source regulators, the ILP incorporated significantly more target submodules than the PathLinker *k=100* network (46 versus 32). This enabled the ILP network to better predict kinase targets. For example, by incorporating more submodules into the network, the ILP network captured 20% more known PKA targets [19, 20] in submodules connected to PKA subunits and harboring the known PKA recognition motif.

The ILP also recovered more SIs than the PathLinker *k=100* network (*k* = 100) (Table 1), which likely enabled it to capture 4X more no-mutant-phenotype submodules by inference (12 compared to 3). This is important, as it allows the method to capture submodules for which no phenotype exists. A key example are submodules downstream of Cdc28: the ILP network linked 152 phospho-peptides with the Cdc28 recognition motif to Cdc28. These peptides are significantly enriched for known Cdc28 targets and further enriched for peptides that show a phosphorylation defect when Cdc28 is inhibited (see main text and Fig 4). The PathLinker *k=100* network captured neither Cdc28 nor its downstream submodules.

Finally, we noticed that the PathLinker paths did not conform to the typical signaling pathway topology. Parts of the PathLinker network include numerous short loops (S2 Fig A), leading to an unusual degree distribution in which some nodes had hundreds of edges (S2 Fig B, C). While in theory this organization could represent feedback, the nodes participating in the looping were often not kinases (*e.g.* the node with the highest degree in the Pde2 *k=200* network is Rpo21, the largest subunit of RNA Pol II which is not a phospho regulator). The unusual topology is likely due to PathLinker’s inability to work on undirected input data. Running PathLinker on undirected PPI data requires expanding an undirected edge into a pair of directed edges. Although each of PathLinker’s source-target paths is a directed, simple path, the union of these paths does not constrain the edge directions. One of its *k* paths can use an edge in one direction and another path can use the same edge in another direction, creating these short loops. This is in contrast to the ILP, which assigns a unique direction to the undirected edges it includes in its paths. Taken together, we show that our ILP-based method outperforms other methods in identifying known regulators and discovering new regulator-target relationships in a dynamic signaling network.

**References**

1. Rose CM, Venkateshwaran M, Volkening JD, Grimsrud PA, Maeda J, Bailey DJ, et al. Rapid phosphoproteomic and transcriptomic changes in the rhizobia-legume symbiosis. Mol Cell Proteomics. 2012;11(9):724-44. doi: 10.1074/mcp.M112.019208. PubMed PMID: 22683509; PubMed Central PMCID: PMCPMC3434772.

2. Hebert AS, Richards AL, Bailey DJ, Ulbrich A, Coughlin EE, Westphall MS, et al. The one hour yeast proteome. Mol Cell Proteomics. 2014;13(1):339-47. doi: 10.1074/mcp.M113.034769. PubMed PMID: 24143002; PubMed Central PMCID: PMCPMC3879625.

3. Wenger CD, Phanstiel DH, Lee MV, Bailey DJ, Coon JJ. COMPASS: a suite of pre- and post-search proteomics software tools for OMSSA. Proteomics. 2011;11(6):1064-74. doi: 10.1002/pmic.201000616. PubMed PMID: 21298793; PubMed Central PMCID: PMCPMC3049964.

4. Lee MV, Topper SE, Hubler SL, Hose J, Wenger CD, Coon JJ, et al. A dynamic model of proteome changes reveals new roles for transcript alteration in yeast. Mol Syst Biol. 2011;7:514. doi: 10.1038/msb.2011.48. PubMed PMID: 21772262; PubMed Central PMCID: PMCPMC3159980.

5. Cox J, Hein MY, Luber CA, Paron I, Nagaraj N, Mann M. Accurate proteome-wide label-free quantification by delayed normalization and maximal peptide ratio extraction, termed MaxLFQ. Mol Cell Proteomics. 2014;13(9):2513-26. doi: 10.1074/mcp.M113.031591. PubMed PMID: 24942700; PubMed Central PMCID: PMCPMC4159666.

6. Chasman D, Ho YH, Berry DB, Nemec CM, MacGilvray ME, Hose J, et al. Pathway connectivity and signaling coordination in the yeast stress-activated signaling network. Mol Syst Biol. 2014;10:759. doi: 10.15252/msb.20145120. PubMed PMID: 25411400; PubMed Central PMCID: PMCPMC4299600.

7. Danna E. FM, Gu Z., Wunderling R. Generating Multiple Solutions for Mixed Integer Programming Problems. In: Fischetti M. WDP, editor. Integer Programming and Combinatorial Optimization. 4513. Berlin: Springer; 2007. p. 280-94.

8. Shannon P, Markiel A, Ozier O, Baliga NS, Wang JT, Ramage D, et al. Cytoscape: a software environment for integrated models of biomolecular interaction networks. Genome Res. 2003;13(11):2498-504. doi: 10.1101/gr.1239303. PubMed PMID: 14597658; PubMed Central PMCID: PMCPMC403769.

9. Gobbi A, Iorio F, Dawson KJ, Wedge DC, Tamborero D, Alexandrov LB, et al. Fast randomization of large genomic datasets while preserving alteration counts. Bioinformatics. 2014;30(17):i617-23. doi: 10.1093/bioinformatics/btu474. PubMed PMID: 25161255; PubMed Central PMCID: PMCPMC4147926.

10. Tuncbag N, Gosline SJ, Kedaigle A, Soltis AR, Gitter A, Fraenkel E. Network-Based Interpretation of Diverse High-Throughput Datasets through the Omics Integrator Software Package. PLoS Comput Biol. 2016;12(4):e1004879. doi: 10.1371/journal.pcbi.1004879. PubMed PMID: 27096930; PubMed Central PMCID: PMCPMC4838263.

11. Bailly-Bechet M, Borgs C, Braunstein A, Chayes J, Dagkessamanskaia A, Francois JM, et al. Finding undetected protein associations in cell signaling by belief propagation. Proc Natl Acad Sci U S A. 2011;108(2):882-7. doi: 10.1073/pnas.1004751108. PubMed PMID: 21187432; PubMed Central PMCID: PMCPMC3021011.

12. Ritz AP, C. Tegge, A. Sharp, N. Simmons, K. Powell, A. Kale, S. Murali, TM. Pathways on demand: automated reconstruction of human signaling networks. npj Systems Biology and Applications. 2016.

13. Haveliwala TH. Topic-Sensitive PageRank: A context-sensitive algorithm for web search. Trans Knowl Data Eng 2003;15:784-96.

14. Grau J, Grosse I, Keilwagen J. PRROC: computing and visualizing precision-recall and receiver operating characteristic curves in R. Bioinformatics. 2015;31(15):2595-7. doi: 10.1093/bioinformatics/btv153. PubMed PMID: 25810428; PubMed Central PMCID: PMCPMC4514923.

15. Davis JG, M. The relationship between precision-recall and ROC curves. Proceedings of the 23rd Interanational Conference on Machine Learning; New York, USA: ACM; 2006.

16. Cherry JM, Hong EL, Amundsen C, Balakrishnan R, Binkley G, Chan ET, et al. Saccharomyces Genome Database: the genomics resource of budding yeast. Nucleic Acids Res. 2012;40(Database issue):D700-5. doi: 10.1093/nar/gkr1029. PubMed PMID: 22110037; PubMed Central PMCID: PMCPMC3245034.

17. de Nadal E, Posas F. Multilayered control of gene expression by stress-activated protein kinases. EMBO J. 2010;29(1):4-13. doi: 10.1038/emboj.2009.346. PubMed PMID: 19942851; PubMed Central PMCID: PMCPMC2808381.

18. Tiger CF, Krause F, Cedersund G, Palmer R, Klipp E, Hohmann S, et al. A framework for mapping, visualisation and automatic model creation of signal-transduction networks. Mol Syst Biol. 2012;8:578. doi: 10.1038/msb.2012.12. PubMed PMID: 22531118; PubMed Central PMCID: PMCPMC3361003.

19. Stark C, Breitkreutz BJ, Reguly T, Boucher L, Breitkreutz A, Tyers M. BioGRID: a general repository for interaction datasets. Nucleic Acids Res. 2006;34(Database issue):D535-9. doi: 10.1093/nar/gkj109. PubMed PMID: 16381927; PubMed Central PMCID: PMCPMC1347471.

20. Sharifpoor S, Nguyen Ba AN, Youn JY, van Dyk D, Friesen H, Douglas AC, et al. A quantitative literature-curated gold standard for kinase-substrate pairs. Genome Biol. 2011;12(4):R39. doi: 10.1186/gb-2011-12-4-r39. PubMed PMID: 21492431; PubMed Central PMCID: PMCPMC3218865.

21. Wu X, Chi X, Wang P, Zheng D, Ding R, Li Y. The evolutionary rate variation among genes of HOG-signaling pathway in yeast genomes. Biol Direct. 2010;5:46. doi: 10.1186/1745-6150-5-46. PubMed PMID: 20618989; PubMed Central PMCID: PMCPMC2914728.

22. Robinson MD, Grigull J, Mohammad N, Hughes TR. FunSpec: a web-based cluster interpreter for yeast. BMC Bioinformatics. 2002;3:35. PubMed PMID: 12431279; PubMed Central PMCID: PMCPMC139976.

23. Hohmann S. Control of high osmolarity signalling in the yeast Saccharomyces cerevisiae. FEBS Lett. 2009;583(24):4025-9. doi: 10.1016/j.febslet.2009.10.069. PubMed PMID: 19878680.

24. Saito H, Posas F. Response to hyperosmotic stress. Genetics. 2012;192(2):289-318. doi: 10.1534/genetics.112.140863. PubMed PMID: 23028184; PubMed Central PMCID: PMCPMC3454867.
