# Supplementary figures and images for: Network inference reveals novel connections in pathways regulating growth and defense in the yeast salt response
Source: PLoS Comput Biol. 2018 May 8;13(5):e1006088. doi: 10.1371/journal.pcbi.1006088 (PMC5940180; doi:10.1371/journal.pcbi.1006088)

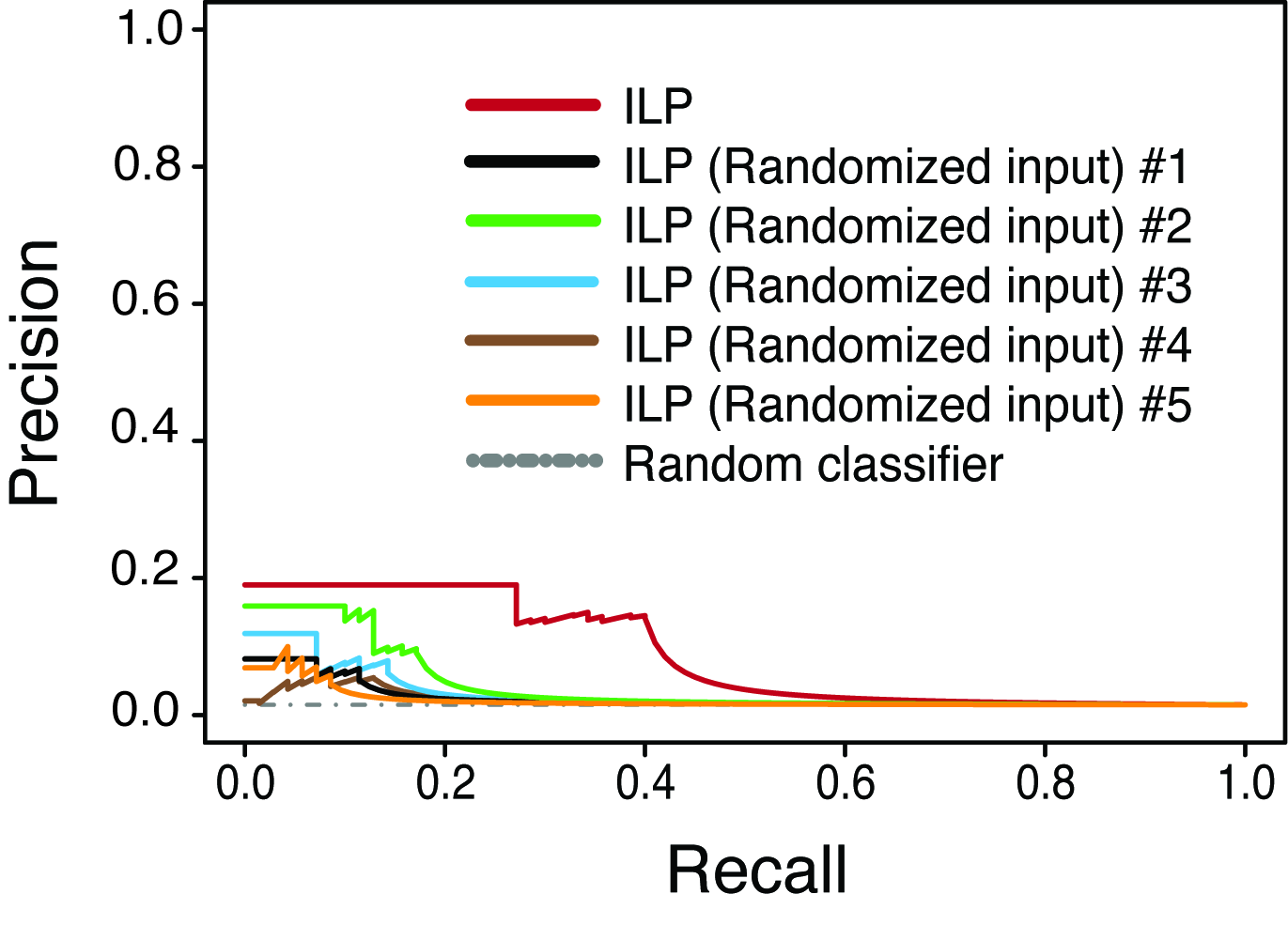

Supplement: S1 Fig — As shown in Fig 2B, the ILP from real data had significantly higher precision and recall of a list of true positive regulators activated by NaCl. The ILP networks from scrambled PPI data also showed significantly lower enrichment for kinases (P from 3.5x10^-12^ to 3x10^-08^) compared to the real ILP network (P = 3.5x10^-36^), and lower enrichment for proteins that interact with Hog1 or are in the HOG pathway (P from 9.1x10^-07^ to 0.07) compared to the real ILP network (P = 8.4x10^-26^). We also compared the collection of SIs identified from real PPI data versus SIs identified from 1,000 scrambled PPI networks. Analyzing the collection of SIs identified in each case, none of the 1,000 randomized trials reached the same precision or recall as real data. We also scored the fraction of times that Hog1 was connected to Hog1-dependent submodules. In the network generated from real PPI data, Hog1 was an SI to three submodules; two of these (67%) matched submodules whose phospho-motifs matched the known Hog1 specificity. In 88% of the randomized trials, Hog1 was never matched to a submodule with the known Hog1 specificity. In 3% of randomized trials, Hog1 was matched to 3 submodules but only 1 matched Hog1 specificity. Thus, while some of the ILP network features are influenced by structure in the PPI background network, many of the insights uncovered here are due to real biology and not simply underlying network structure. (TIF) [file pcbi.1006088.s002.tif]

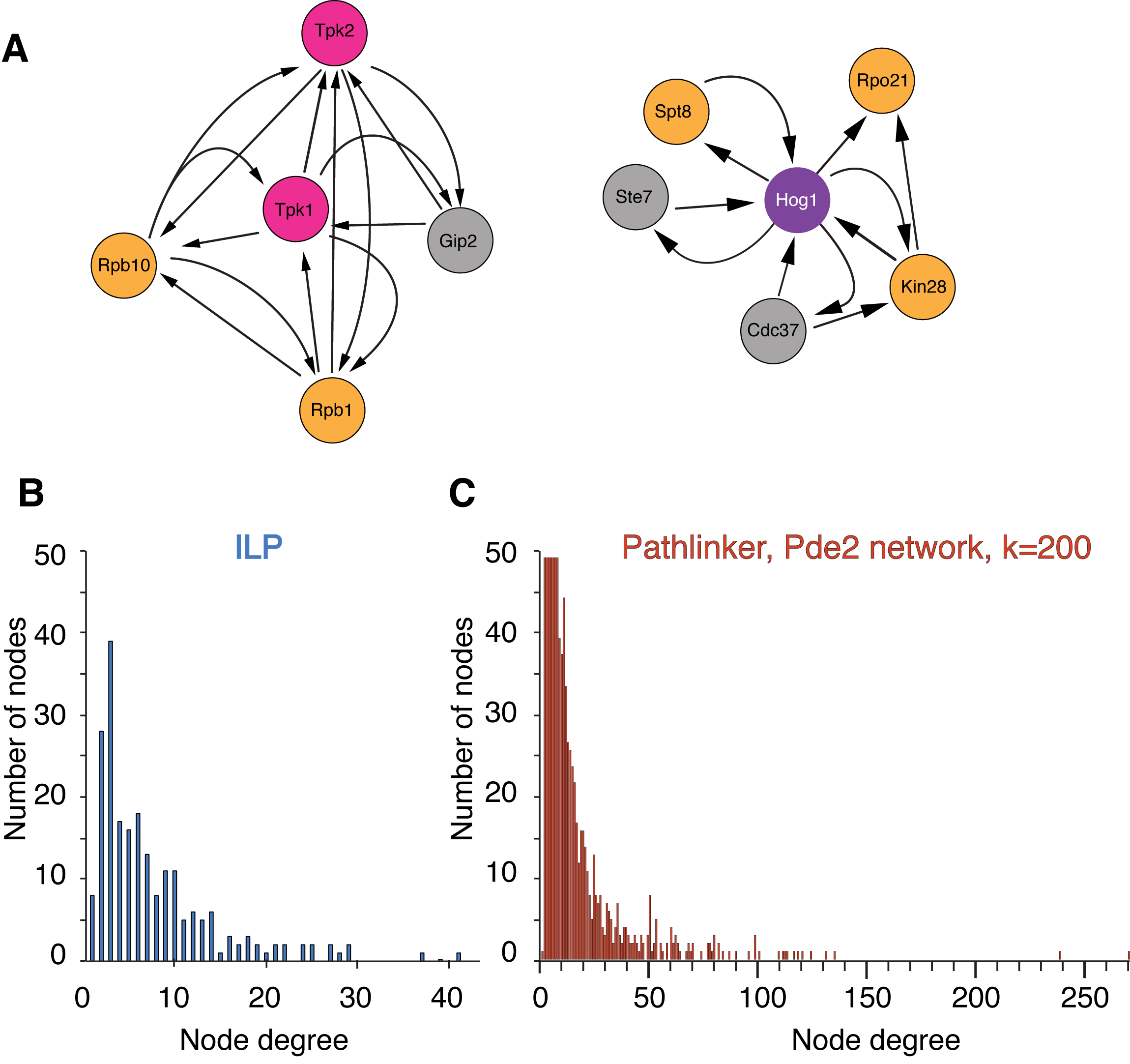

Supplement: S2 Fig — A) Examples of looping seen in the PathLinker k = 200 network inferred for the Pde2 source. These examples were manually chosen for nodes connected to Tpk1 or Hog1. B-C) Degree distribution for the B) ILP (75% confidence) network and the C) Pathlinker k = 200 Pde2 network. PKA subunits (pink nodes), Hog1 (purple node), and proteins involved in transcriptional regulation (orange node). (TIF) [file pcbi.1006088.s003.tif]
